# QsRNA-seq: a method for high-throughput profiling and quantifying small RNAs

**DOI:** 10.1186/s13059-018-1495-0

**Published:** 2018-08-14

**Authors:** Alla Fishman, Dean Light, Ayelet T. Lamm

**Affiliations:** Faculty of Biology, Technion - Israel Institute of Technology, Technion City, 32000 Haifa, Israel

**Keywords:** High-throughput sequencing, miRNA, *C. elegans*

## Abstract

The ability to profile and quantify small non-coding RNAs (sRNAs), specifically microRNAs (miRNAs), using high-throughput sequencing is challenging because of their small size. We developed QsRNA-seq, a method for preparation of sRNA libraries for high-throughput sequencing that overcomes this difficulty by enabling a gel-free separation of fragments shorter than 100 nt that differ only by 20 nt in length. The method allows the use of unique molecular identifiers for quantification and is more amenable to automation than gel-based methods. We show that QsRNA-seq gives very accurate, comprehensive, and reproducible results by looking at miRNAs in *Caenorhabditis elegans* embryos and larvae.

**Electronic supplementary material:**

The online version of this article (10.1186/s13059-018-1495-0) contains supplementary material, which is available to authorized users.

## Background

Small RNAs (sRNAs) are non-coding RNA molecules, 20–30 nucleotide (nt) long, that impact diverse biological events through the control of gene expression and genome stability. During the last decade, sRNAs emerged as central players in the regulation of gene expression in all kingdoms of life [1], and they have been shown to regulate virtually all cellular processes. There are several classes of sRNA [2], with Dicer-generated microRNAs (miRNAs) being the most extensively studied [3].

High-throughput sequencing (HTS) is currently the method of choice for identifying and analyzing the cellular repertoire of RNAs because it allows one to investigate the entire transcriptome in an unbiased way. While preparation of mRNA and DNA libraries for HTS has become a routine procedure, preparation of sRNA libraries remains technically challenging. Most protocols for generation of sRNA libraries require the sRNA molecules to be ligated from both sides (5′ and 3′) to oligonucleotide adapters that contain the sequencing primers, reverse transcribed for complementary DNA (cDNA) generation, and amplified by polymerase chain reaction (PCR) [4]. The first challenge that arises at the very beginning of library preparation is separation of the sRNAs from other RNA species close in size, in particular transfer RNA (tRNA). Omission of this step results in sRNA libraries that are highly contaminated. In addition, the adapters readily ligate to each other instead of to the RNA, and this product (adapter dimer), if not removed, is preferably amplified by PCR, resulting in null sequences in the HTS data. Because of the small size difference between sRNAs and tRNA and between the sRNA-ligated product and adapter dimer, it is very difficult to separate them, decreasing library quality. Denaturing polyacrylamide gel (PAGE), commonly used for size-based separation of small fragments, not only is time consuming and requires expertise, but also results in a significant loss of the product and eliminates the possibility of automating the pipeline. Solid-phase reversible immobilization (SPRI) on magnetic beads [5, 6] is widely used for nucleic acid separation based on size; however, this method is not able to discriminate between fragments shorter than 100 nt and thus is not applicable for preparation of sRNA libraries.

The implementation of HTS for sRNA profiling is also hindered by the inability to reliably quantify the output data. Small RNA library construction for HTS involves a PCR amplification step that is prone to bias. Because PCR is not a linear process, it quickly reaches plateau, distorting differences in expression. Moreover, PCR efficiency depends on the length of the fragment and on its sequence; variations in base composition might lead to preferred template-specific amplifications [7]. Other biases can be introduced during library preparation including ligation bias during adapter ligation (reviewed in [8]). HTS-based miRNA expression data is thus not regarded as quantitative, and other techniques, mostly quantitative real-time PCR (qPCR) and miRNA microarrays, are used instead for quantification of individual miRNA and for large-scale studies, respectively. While these two assays are accurate and sensitive, both require prior knowledge and accurate annotation of the miRNA sequence tested and are not applicable to discovering novel miRNAs.

PCR-derived artifacts can be corrected by counting absolute numbers of molecules using unique molecular identifiers (UMIs) [9] . This method allows distinguishing between original copies of the sRNA present in cells and their amplification products by marking, prior to the amplification step, each molecule in a population by attaching a UMI, a short random sequence. Following amplification, each one of the UMIs attached to an original copy of the molecule will be observed multiple times; however, the original copy number of a molecule can be determined simply by counting each UMI only once upon analysis of HTS sequencing data. Thus, UMIs in the library act as a molecular memory for the number of molecules in the starting sample. In addition, it was shown that using UMIs reduces ligation bias by randomizing the adapter sequences at the ligation junction [8, 10]. Surprisingly, this method is rarely used in generation of sRNA libraries.

Here, we present QsRNA-seq, a novel method for preparation of sRNA libraries for HTS sequencing that overcomes the above-mentioned shortcomings. Our protocol comprises two innovations: (1) gel-less size-based separation of fragments shorter than 100 nt, differing in length by 20 nt or more, and (2) use of UMIs to enable quantification of sRNA expression data.

## Results

### A novel method for separating nucleic acids shorter than 100 nucleotides

**Fig. 1:**
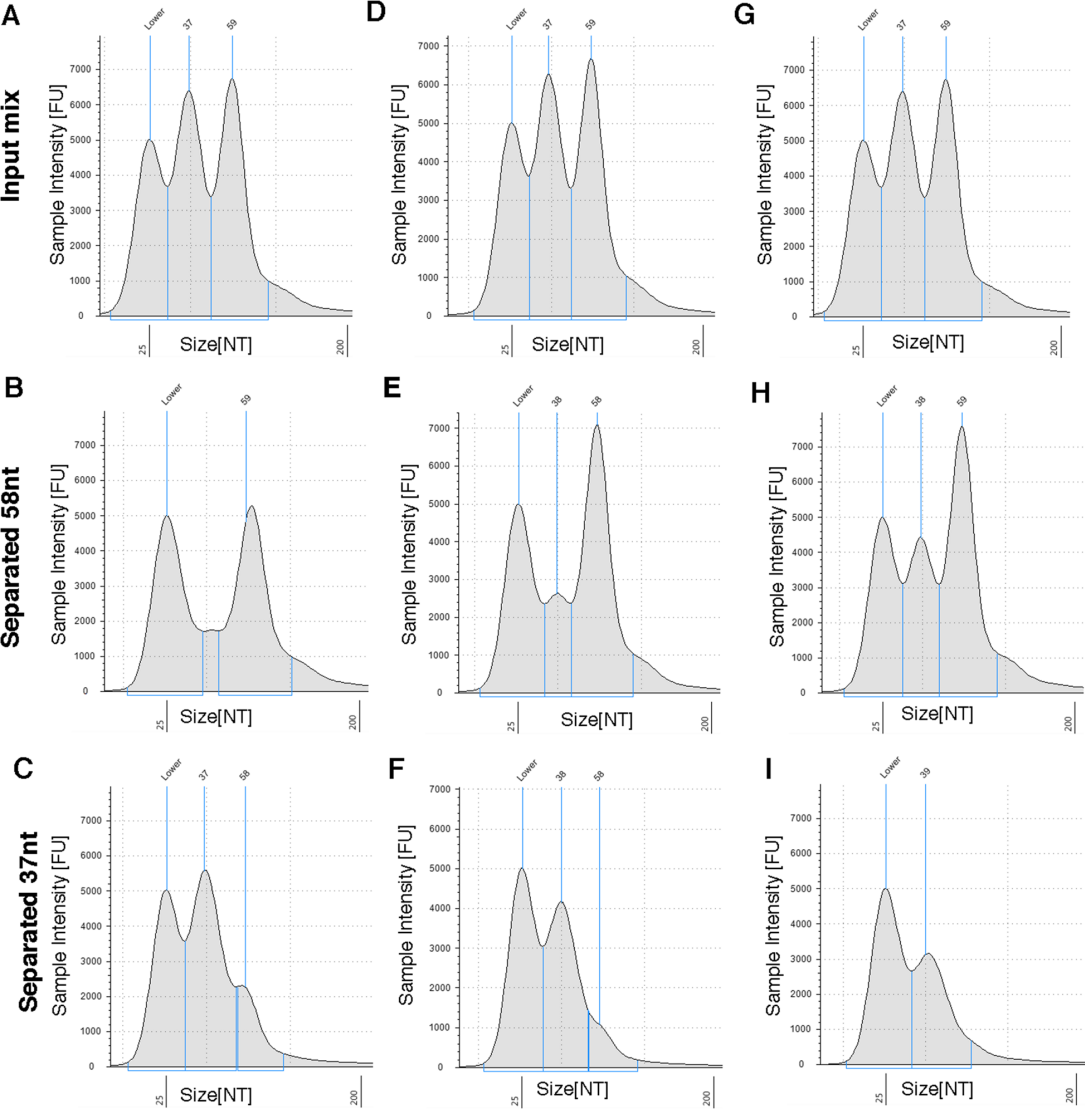
Separation of 37-nt and 58-nt fragments. TapeStation traces of an input mix of two single-stranded DNA oligonucleotides, 37 nt and 58 nt, separated by double-sided size selection on SPRI beads. The first size-selection step was performed at three different isopropanol conditions, 38% (**a**, **b**, **c**), 41% (**d**, **e**, **f**), and 44% (**g**, **h**, **i**). Input oligonucleotide mixtures for each concentration are presented in (**a**, **d**, **g**). Eluates of the first size-selection step using each isopropanol concentration are presented in (**b**, **e**, **h**). Eluates of the second size selection using each isopropanol concentration are presented in (**c**, **f**, **i**). Peak sizes and corresponding fragments areas are marked in *blue*; the *left peak* titled “Lower” is a 25-nt size marker

Most of the difficulties in sequencing and quantifying sRNAs derive from their small size. To separate them from tRNA and to separate ligation products from adapter dimers, a technique is required that will allow simple and reliable separation based on a fragment size ranging between 20 and 100 nt. To achieve this, we modified SPRI [5, 6], a size-selection method based on a non-specific reversible binding of nucleic acid molecules to carboxyl groups-coated magnetic beads in the presence of a “crowding agent” such as polyethylene glycol (PEG). As the efficiency of binding is dependent on the length of the fragment and the concentration of the crowding agent, it is possible to separate two fragments of different lengths. It is well known that adding alcohol, another crowding agent, to PEG modifies the range of bound fragment sizes, allowing binding of molecules as short as 18 nt to the magnetic beads. Our hypothesis was that by adjusting the concentration of isopropanol added to PEG, we would be able to achieve separation of molecules shorter than the 100-nt threshold [11]. Therefore, we prepared a series of SPRI-based size-selection solutions, all having the same concentration of PEG (7.5%) but different concentrations of isopropanol, ranging from 32% to 54.5%, and tested their ability to promote binding of synthetic single-stranded DNA oligonucleotides of different lengths to the beads (see Methods). The oligonucleotides sizes, ranging from 19 to 66 nt, were chosen to cover the separation steps needed for sRNA library preparation, namely separation of sRNA from tRNA, separation of 3’ adapter-ligated sRNA from free 3’ adapter, and separation of 3′,5′ adapter-ligated sRNA from adapter dimer. The binding efficiency was calculated by the ratio of oligonucleotide quantities in the eluent versus the input using a fluorometer. The results of the experiment are summarized in Table 1. As hypothesized, increasing the concentration of isopropanol leads to an increase in binding efficiency. Moreover, for each oligo length tested, we determined a condition resulting in its significant binding (> 40%) to the beads, while oligos shorter by 20 nt bound poorly (< 5%). We next tested the feasibility of using these conditions to separate two oligonucleotides, 37 nt and 58 nt, differing in length by 21 nt. We used two-step size selection on the SPRI beads, by (1) binding the longer fragment to the beads and collecting the unbound material containing the shorter fragments and (2) adding a second batch of beads and isopropanol, and adjusting the conditions (based on Table 1) to allow complete binding of the shorter fragment. Eluting the first and second batch of beads isolated the longer and shorter fragments, respectively. For isolating the 58-nt oligos, we used three different concentrations of isopropanol at the first step: 38%, 41%, and 44%. The supernatant, containing the unbound shorter oligonucleotide, was transferred to a new tube, and the second step was performed at 54.5% isopropanol to allow maximal recovery of the 37-nt oligos. The input mixture and eluates from each size-selection step were analyzed using TapeStation (see Methods and Fig. 1). Binding efficiencies were consistent with those determined using a single oligo (Table 1). Using 38% isopropanol at the first step of the size selection recovered around two thirds of the 58-nt input material with minor leftovers of the 37-nt oligos (Fig. 1a, b), while the second step size selection resulted in nearly complete recovery of the 37-nt oligos but a third of the input material of the 58-nt oligos (Fig. 1c). Using 44% isopropanol resulted in a mirror picture: a complete recovery of the 58-nt oligos with a noticeable fraction of 37-nt oligos (Fig. 1g, h) by the first step, while the second step size selection yielded a third of the 37-nt input oligos with almost no 58-nt oligos (Fig. 1i). In-between results were observed when using 41% isopropanol (Fig. 1d, e, f). We conclude that by using the concentrations of isopropanol presented in Table 1, it is possible to separate between two short nucleic acids differing in length by 20 nt with high recovery.

**Table 1:** Binding efficiencies of single-stranded DNA (ssDNA) oligonucleotides to SPRI beads at varying isopropanol concentrations

Oligo size (nt)	Isopropanol concentration (%)								
	30	32	35	38	41	44	48	51	54.5
66	21	56	74	87	97	100	ND	ND	ND
58	ND	4	8	42	80	89	ND	ND	ND
44	ND	2	3	13	40	59	75	ND	ND
37	ND	ND	1.5	4	11	19	45	80	ND
30	ND	ND	ND	ND	4	5	20	40	61
21	ND	ND	ND	ND	ND	2	4	7	15
19	ND	ND	ND	ND	ND	1	3	3	5

100 ng ssDNA oligonucleotides were brought to a total volume of 50 μl with H_2_O, and appropriate amounts of SPRI beads (in 20% PEG, 2.5 M NaCl) and 100% isopropanol were added to obtain the desired concentrations (for added quantities see Methods). Oligonucleotides bound to beads were next separated, washed, and eluted. Oligonucleotide quantities in the eluate and in the input solution were determined by fluorometer. Binding efficiency was calculated as the percentage of eluted ssDNA from the input quantity. Each data point represents an average of at least three separate experiments.

*ND* not determined

### QsRNA-seq: a method for preparation of small RNA libraries

**Fig. 2:**
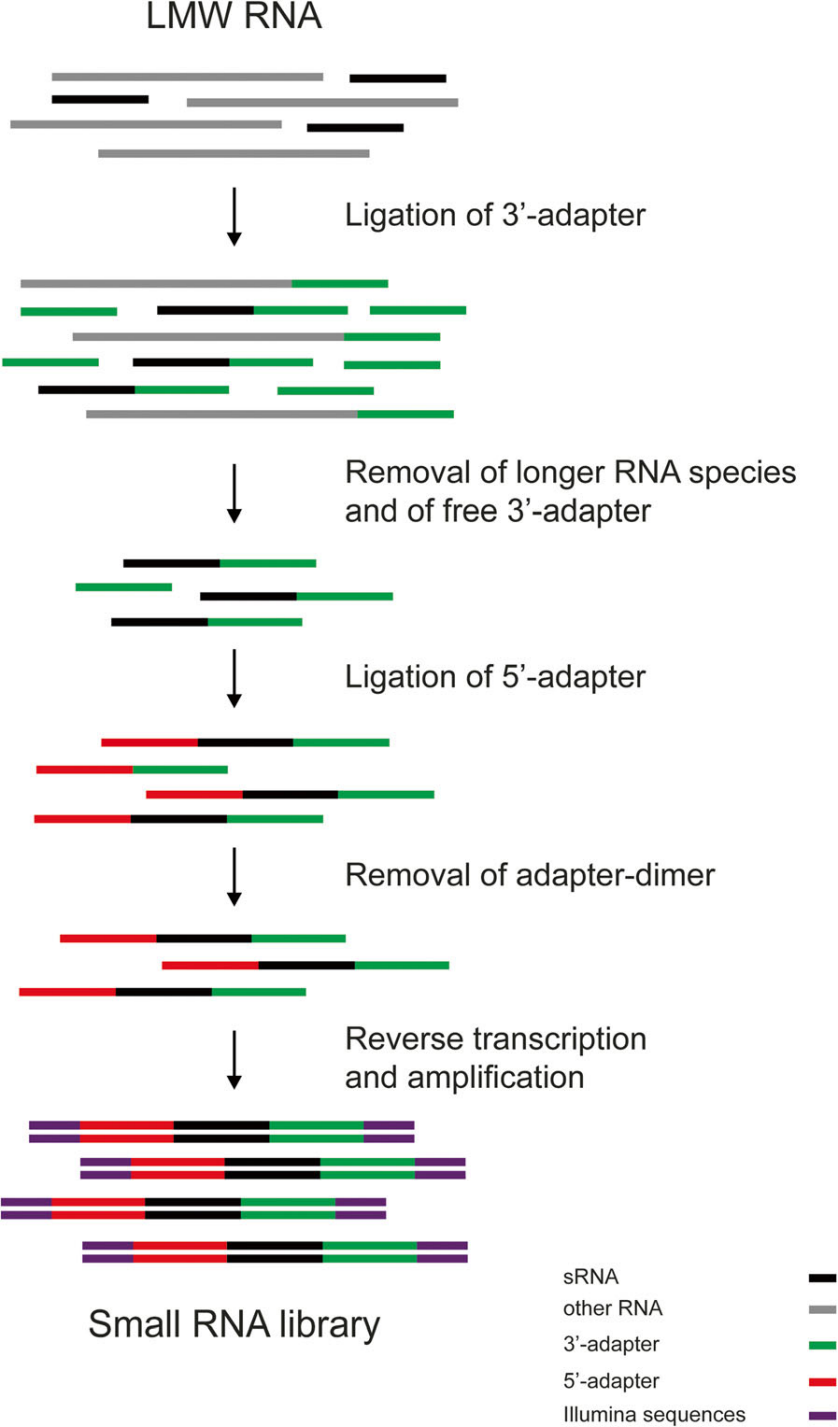
QsRNA-seq library preparation scheme. A general scheme for preparation of sRNA library for high-throughput sequencing. Low molecular weight (*LMW*) RNA fraction is ligated to 3′ adapter. Next, the 3′-ligated sRNA is separated first from longer RNA species (tRNA, mRNA, etc.) and then from the remaining free 3′ adapter. 3′-ligated sRNA is then ligated to 5′ adapter possessing UMI. In the next step, the 3′-5′-ligated sRNA is separated from free 5′ adapter and from 3′-5′ adapter dimer and subjected to reverse transcription and PCR amplification. All the separation steps of the protocol are performed by size selection using SPRI paramagnetic beads

We next designed a new protocol for preparation of sRNA libraries for HTS, utilizing the separation method we developed. The protocol, named QsRNA-seq, is presented in Fig. 2 (for the detailed protocol see Additional file 1). The protocol, based on [12, 13], implements two ligation steps: (1) ligation of pre-adenylated 3′ adapter without ATP and (2) ligation of 5′ adapter containing a 4-nt barcode to allow multiplexing. Three size-separation steps on SPRI paramagnetic beads are performed during the protocol to obtain only the required RNA molecules: (1) separation of 3′ adapter-ligated sRNA from longer RNAs (mainly tRNA), (2) separation of 3′ adapter-ligated sRNA from free 3′ adapter, and (3) separation of 3′,5′ adapter-ligated sRNA from adapter dimer and free 5′ adapter (for sizes of fragments see Additional file 2: Table S1).

The QsRNA-seq protocol is based on the classic sRNA sequencing PAGE-based method [12]. While the PAGE-based method is reliable and has been used in many studies, it is tedious and results in a very low yield. To estimate the efficiency of QsRNA-seq, we prepared libraries from 20 pmol of 22-nt-long synthetic RNA oligo by both methods (QsRNA-seq and the PAGE-based method, two replicas each) and directly quantified the obtained 3′,5′-ligated sRNA using Bioanalyzer. The average yield from QsRNA-seq was about five times higher than that from the PAGE-based method (Additional file 2: Figure S1). Moreover, both replicas generated by QsRNA-seq in terms of quantities were very similar, while the PAGE-generated replicas were different and needed PCR optimization. In addition, reducing the starting material by 10-fold when generating libraries by QsRNA-seq yielded a similar outcome (see below). Therefore, we conclude that QsRNA-seq is much more efficient and robust than the standard PAGE-based method.

To further test the quality of QsRNA-seq-generated libraries, we used RNA extracted from *Caenorhabditis elegans* embryos because the PAGE-based method was established in *C. elegans* and has been widely used to study sRNA in this organism. From the same RNA we generated three technical replicas using the classic PAGE-based method with three different barcodes and three technical replicas using QsRNA-seq with the same barcode set. In all six libraries, the fraction of tRNA, which is very close in size to the sRNAs, was minimal (Additional file 2: Table S2). The small nucleolar RNA (snoRNA) fraction was also very low, while the ribosomal RNA (rRNA) fraction was relatively high, ranging from 20% to 54%. rRNAs are larger in size than tRNA and both are very abundant, suggesting that the high fraction of rRNA is a result of RNA degradation and the quality of the RNA. Indeed, earlier generated libraries (8N_AAGA and PAGE based, Additional file 2: Table S2) contain a lower fraction of rRNA. Comparing the two methods, we found a very significant correlation in miRNA expression (see below and Additional file 2: Figure S5) and very similar read length distribution (see below, Additional file 2: Figure S11) and first nucleotide preference (Additional file 2: Figure S9). Collectively, our results indicate that using QsRNA-seq generates sRNA libraries of equivalent quality and higher yield than those obtained using the classic PAGE-based method.

Small RNA library generation is vulnerable to many biases (reviewed in [8]). Ligation bias, which results from adapter preference, is one of the major biases in library generation. It was shown that adding a UMI to each RNA molecule can reduce the ligation bias [10] and that adding the UMI before the PCR step can correct for PCR-induced artifacts and enable quantification. Therefore, we also used 5′ adapters that contain eight random nucleotides that provide UMIs in our QsRNA-seq method. After PCR amplification, we considered identical sRNAs with the same UMI as an amplification product and merged them to one sequence (known as collapsing). Comparing libraries generated from the same RNA with and without the UMI with the same set of barcodes, we found that adding the UMI significantly reduced the biases. Evaluating the expression of miRNAs in RNA extracted from *C. elegans* embryos, we found that more than half of the miRNAs have an improvement in the adapter bias when UMIs are added (Additional file 2: Figure S2, 0Nvs0N compared to 8Nvs8N). After collapsing the samples with the UMI, two thirds of the miRNAs had a reduction in library construction biases with a 30% average bias reduction (Additional file 2: Figure S2, 0Nvs0N compared to 8Ncolvs8Ncol). In addition, adding a UMI seems to improve miRNA quantification (see also the discussions in subsequent sections).

### QsRNA-seq can evaluate miRNA abundance and expression changes accurately

To test the ability of QsRNA-seq to detect sRNAs, we used QsRNA-seq on RNA extracted from wild-type *C. elegans* synchronized to the embryo or the L4 larval stage and on total RNA obtained from human brain. Human brain total RNA was chosen because miRNAs constitute most of the sRNAs in this sample; thus, we expected it to result in a very uniform library. In contrast, *C. elegans* contains many types of sRNA, including miRNAs, primary and secondary endogenous small interfering RNAs (siRNAs), and piwi-interacting RNAs (piRNAs). We generated three independent biological samples from each *C. elegans* developmental stage, embryo and L4, as biological replicas. RNA extracted from one sample from each stage was also subjected to three independent library preparations, as technical replicas, and was also used to prepare three technical replica libraries having no UMI in the 5′ adapter (0 N). All library preparations resulted in very clean products ready for sequencing with a negligible ratio of adapter dimer containing no product (less than 2% of the total reads in each library; see the examples in Additional file 2: Figure S3 and the library information in Additional file 2: Table S3).

To evaluate the quality of the QsRNA-seq output sequences, we aligned the generated sequences to all annotated miRNAs (both miRNA and miRNA*) in *C. elegans*, miRBase WBcel235, or in human, miRBase GRCh38. In the *C. elegans* sample, all the annotated miRNAs (100%) were present in our samples by at least one strand (3P or 5P), while 97% of all microRNAs had coverage for both strands. Even rare miRNAs such as lsy-6, which is expressed in only one pair of neurons in the *C. elegans* head, were present [14] (Additional file 3: Table S4). QsRNA-seq allows extensive multiplexing of the samples before amplification, which can reduce significantly the amount of starting material required. However, even without multiplexing, reducing the starting material by 10-fold, from 1 μg to 100 ng, produced nearly identical results (Additional file 2: Figure S4). In human brain, the coverage was somewhat lower, with alignment to 80% of annotated miRNAs (Additional file 4: Table S5). The difference probably derives from the large number of samples that we generated from whole worms at two developmental stages while we only generated one sample from human cells from a specific tissue. However, miRNAs known to be enriched in human brain, for example, let-7 family, mir-9, mir-26a, and others [15], were very abundant in our libraries.

We also compared miRNA expression in the libraries generated by the PAGE-based method and QsRNA-seq method from the same RNA without UMIs and found a very significant correlation (Pearson correlation *R*^2^ = 0.88, *p* value <  2.2e-16, Additional file 2: Figure S5).

**Fig. 3:**
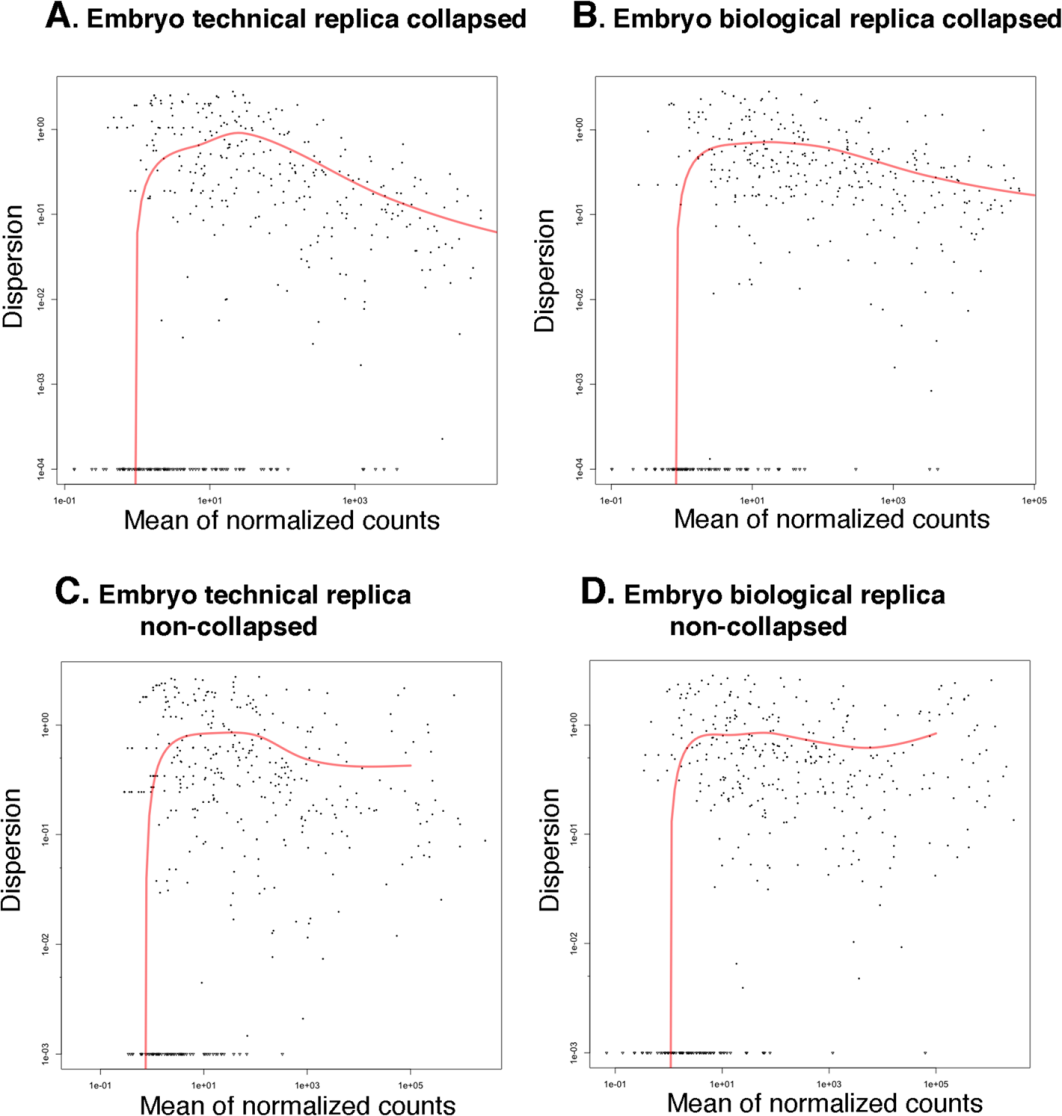
Variance between collapsed and non-collapsed biological and technical replica samples at embryo stage is low. Dispersion plots generated by the DESeq package in R using the estimateDispersions function. Sequences aligned to each miRNA were counted and variance of samples was estimated. Each *dot* in the plot represents variance between samples for specific miRNA counts. *Y*-axis presents a dispersion value, which is the difference between samples squared. For example, a miRNA whose expression in different replicas differs by 10% will have a dispersion value of 0.01. *X*-axis is the mean of normalized counts. All plots are samples generated from embryo developmental stage: **a** technical replica samples dispersion estimated with collapsed reads, **b** biological replica samples dispersion estimated with collapsed reads, **c** technical replica samples dispersion estimated with non-collapsed reads, and **d** biological replica samples dispersion estimated with non-collapsed reads

To further assess the consistency of the method, we evaluated the dispersion of miRNA expression between the replica samples, biological and technical, collapsed and non-collapsed. As expected and consistent with our observation that using collapsing reduces library preparation biases, the collapsed replica exhibited lower dispersion rates than the corresponding non-collapsed replica, for both biological and technical replica types (Fig. 3, Additional file 2: Figure S6). For example, comparing the dispersion of the biological samples at embryo stage between collapsed reads and non-collapsed reads (Fig. 3b, d), we observed at high normalized counts (> e + 03) that the dispersion in the collapsed samples ranges between around e-0.5 and e-0.8, whereas the non-collapsed counts range between e-0.125 and e-0.22. Moreover, collapsed count dispersion decreases as the mean of the normalized counts increases, thus confirming our assumption that collapsing will tend to reduce statistical errors more significantly when dealing with larger counts.

The increase in variation between samples, before and after collapsing, correlates with the abundance of miRNA in the initial samples, due to biased amplification of the abundant miRNA by PCR. While the ratio between the number of reads obtained for a single miRNA by collapsing and by non-collapsing is not significant for a low abundance miRNA (Additional file 2: Figure S7A), it increases drastically in direct relation to miRNA abundance. Interestingly, the ratio becomes significant (above twofold) when the initial expression level of a miRNA rises above 100 copies (Additional file 2: Figure S7A). Despite this, the higher read number obtained by non-collapsing does not affect the global picture of differential expression between the L4 and embryo stages, i.e., whether a miRNA is upregulated or downregulated (Additional file 2: Figure S8). However, non-collapsing augments the magnitude of L4/embryo expressional fold change for a subset of miRNAs (Additional file 2: Figure S7B).

### The 23-nt-long small RNAs are enriched in L4 larvae and not in embryos in *C. elegans*

**Fig. 4:**
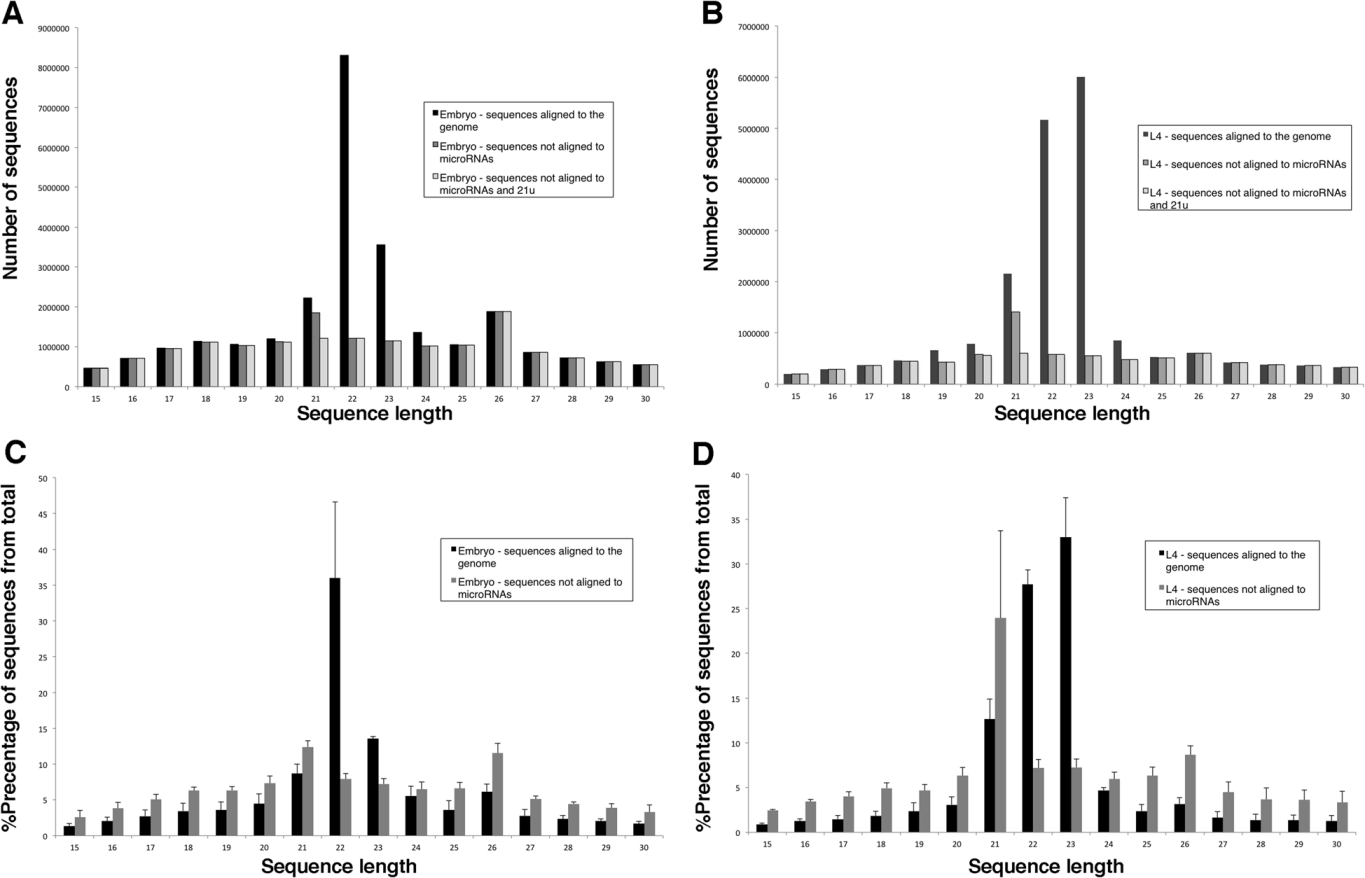
Size distribution of the *C. elegans* sRNA sequences. **a**, **b** Bar charts presenting number of sequences for each sRNA sequence length from 15 to 30 nt. The sequences are from one sample from embryo stage (**a**) or L4 larval stage (**b**). **c**, **d** Bar graphs presenting percentage of sRNAs for each sequence length from 15 to 30 nt from total number of sequences. **c** Average of three biological replicates at embryo stage. **d** Average of three biological replicates at L4 larval stage. Standard deviation is also presented. *Black bars* represent sequences that align to the genome, *dark gray bars* are sequences that did not align to miRNAs from sequences presented in the black bars, *light gray bars* represent sequences from the dark gray bars that also did not align to 21 U sRNAs

Besides miRNAs, which are mostly 22 nt long, *C. elegans* contains many other endogenously generated types of sRNAs [16]. siRNAs, which are very abundant in *C. elegans*, fall into two groups: (1) primary siRNAs, which are 5′ monophosphate and are 26 nt long, and (2) secondary siRNAs, which are 5′ triphosphate and are 21–22 nt long. Another group of sRNAs is the equivalent of piRNAs in *C. elegans*, the 21 U group, which are 21 nt long. To assess whether QsRNA-seq is capable of detecting all sRNA types, we performed length distribution on all the genome-aligned sequences from libraries generated from *C. elegans* embryo and L4 larval stages (Fig. 4). As the libraries were prepared in a way that captures mostly RNAs with 5′ monophosphate and not 5′ triphosphate by direct ligation, we did not expect to have many secondary siRNAs in these samples. Surprisingly, we observed a major difference in sequence length distribution between samples generated from embryos and samples generated from L4 larvae. We found that in embryos the most prominent length of sRNAs is 22 nt, while in L4 larval samples the prominent length is 23 nt (Fig. 4). By removing sequences that aligned to miRNAs and performing a new length distribution, we found that most of the 22-nt- and 23-nt-long sequences are miRNAs (Fig. 4a, b, black bars versus dark gray bars). Similar analysis performed on the 21-nt-long sequences showed that these sequences, detected in all the samples in considerable quantities, are mostly 21 U (Fig. 4a, b). The 26-nt-long sequences predominantly start with a G both in libraries generated by QsRNA-seq and in libraries generated by PAGE (Additional file 2: Figure S9). Thus, the 26-nt peaks are primary siRNAs, which have a preference to 5’ G [17]. Sequences generated from technical replicas or from samples without UMI showed similar length distribution (Additional file 2: Figure S10). The length distribution of samples generated by the PAGE-based method was very similar to that of the samples generated by QsRNA-seq (Additional file 2: Figure S11). However, sequences shorter than 20 nt were almost not present in the PAGE samples because the gel was cut around this length to eliminate non-ligated 3’ adapter. As expected, length distribution performed on the human brain sample showed significant peaks at 21–23 nt, corresponding mainly to miRNAs (Additional file 2: Figure S12).

To evaluate whether QsRNA-seq can also capture 5′ triphosphate secondary siRNAs, which are mainly 22 nt in size, we added a step to the QsRNA-seq protocol, in which RppH enzyme (New England Bioloabs (NEB)) was used to remove two 5′ phosphates to enable ligation to the 5′ adapter. Using the modified protocol, we generated three biological replicas from the embryo stage and three biological replicas from the L4 stage. The length distribution of the sequences was as expected (Additional file 2: Figure S13): in both the embryo and L4 developmental stages most of the sequences were 22 nt long. The portion of miRNAs from these sequences was very low compared to sequencing that captures mainly 5' monophosphate sRNA (Fig. 4, Additional file 2: Figure S13), suggesting that these are mainly secondary siRNAs. In addition, we observed the difference in length distribution of sequences between embryo and L4 in this sequencing method as well; the portion of 23-nt-long sequences is much higher in L4 compared to embryo, and these 23-nt-long sequences are mainly miRNAs (Additional file 2: Figure S13).

**Fig. 5:**
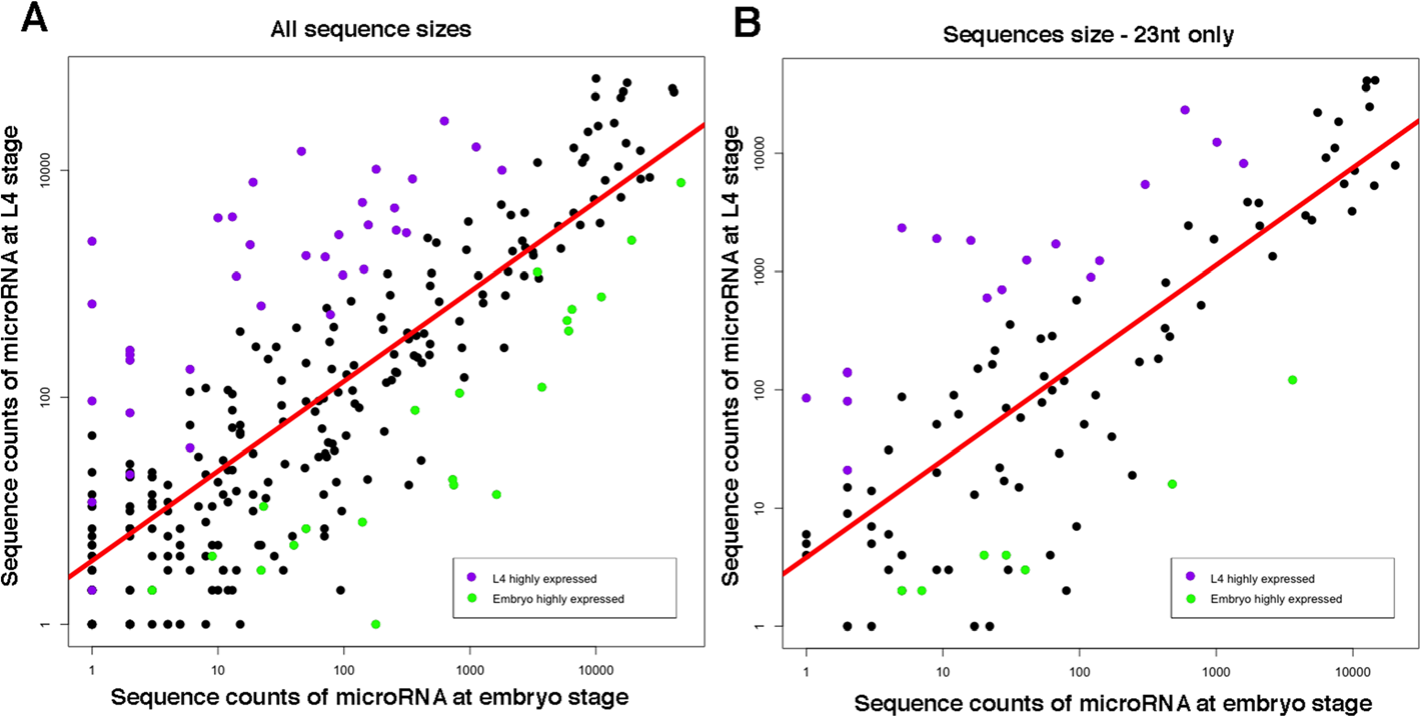
The 23-nt-long miRNAs are predominantly expressed in L4 larval stage. Log scale plots comparing miRNA expression between embryo and L4 developmental stages. **a** All miRNAs, **b** 23-nt-long miRNAs only. Every *dot* in the plot represents a miRNA. *Green dots* indicate miRNAs predominantly expressed at embryo stage; *purple dots* indicate miRNAs predominantly expressed at L4 stage. The *red line* is the regression line for all miRNAs presented in the graph

To further study the difference in length distribution of miRNAs between embryo and L4 samples, we evaluated the miRNA expression changes between embryo and L4 developmental stages and estimated the fold change difference. Selecting miRNAs with at least a fivefold difference and an adjusted *p* value after Benjamini–Hochberg correction < 0.01, we found 30 miRNAs that are predominantly expressed in embryo and 38 miRNAs that are predominantly expressed in L4 (Fig. 5a, Additional file 2: Table S3). Our expression analysis is comparable to published data. For example, our findings that lin-4 family and let-7 family members are predominantly expressed during larval development, and miR-35 family members are predominantly expressed in embryogenesis, are in full concordance with data obtained using northern blot analysis [18, 19]. Our analysis is also comparable with large-scale studies performed either by multiplexed qPCR [20] (82% of predominantly expressed miRNAs in L4 and 100% in embryos were found by our analysis) or by HTS [21] (96% of predominantly expressed miRNAs in L4 and 55% in embryos were found by our analysis). Our analysis was very restrictive — three biological replicas, fivefold or more expression changes, and padj < 0.01 — as compared to the other two analyses, which can explain the discrepancies between the lists.

Interestingly, evaluating miRNA expression using only sequences that are 23 nt long (Fig. 5b), we found a high fraction of 23-nt-long miRNAs that are predominantly expressed in L4 (45%) as compared to miRNA predominantly expressed in the embryo stage (23%) (*p* value < 0.05 by chi-square test). Evaluating miRNA expression using sequences that are 22 nt long, we did not observe a significant difference in the fraction of miRNAs predominantly expressed in L4 versus embryo stage (Additional file 2: Figure S14). Evaluating the portion of highly expressed miRNAs in the L4 developmental stage that are 23 nt long compared to 22 nt long at the samples enriched for secondary siRNAs (Additional file 2: Figure S13), we observed a very similar result with a significant portion of 23-nt-long miRNAs compared to 22-nt-long miRNAs (*p* value < 0.05 by chi-square test), which was not observed at the embryo stage. Thus, we conclude that in *C. elegans* the expression of 23-nt-long miRNAs is developmental stage specific.

## Discussion

Although sRNAs (20–30 nt) constitute a very small fraction of the total cellular RNA, they have a significant role in every aspect of cellular and organismal development and maintenance [1]. Thus, identification, characterization, and quantification of sRNAs are an important part of many studies. miRNAs are also promising clinical diagnostic and predictive biomarkers [22]; in particular, miRNAs circulating in body fluids are attractive candidates to serve as markers in non-invasive “liquid biopsies” [23]. Unfortunately, because of the difficulty in isolating sRNAs from other nucleic acids very close in size, profiling sRNAs using HTS is often avoided. To overcome this problem, we determined the best conditions to separate short nucleic acid fragments using SPRI beads. Our screen resulted in the development of a method to separate fragments shorter than 100 nt differing in length by as few as 20 nt. The method also allows flexibility based on the tradeoff between purity and quantity (e.g., better separation between the fragments but less recovery of the desired fragment). While we concentrated on the binding efficiency of fragments between 19 and 66 nt, for needs of library preparation, binding conditions for fragments ranging between 60 and 100 nt can be easily determined in the same manner for any other purpose.

We developed a new protocol, QsRNA-seq, substituting PAGE size selection by SPRI beads, which resulted in a much quicker, technically easier protocol that enables parallel processing of samples with higher yield. The protocol comprises all the separation steps required for preparing high-quality sRNA libraries for HTS. The 20-nt separation resolution that we gained is sufficient to significantly reduce the two main contaminants of sRNA libraries: tRNAs and adapter dimers. To further avoid adapter dimer contamination, we also include in the protocol a common method of turning a free 3′ adapter into a double-stranded structure by hybridizing the 3′ adapter with a reverse complementary oligonucleotide [24]. Lack of contaminants resulted, as expected, in an impressive sequencing depth. The sequences that were obtained aligned to 97% of the annotated *C. elegans* miRNAs. All other sRNA types known in *C elegans*, such as endogenous primary and secondary siRNA and 21 U-RNA, were present in significant quantities. The presence of short (20–40 nt) degradation products of other RNA species, in particular long and abundant rRNA molecules, cannot be avoided by this method but can be minimized by using high-quality RNA.

While loss of material during QsRNA-seq is significantly reduced since no gel purification is required, an amplification step is still needed to produce enough material for HTS. PCR is usually used for amplification; however, it is not a linear process and is not free of biases [7]. In contrast to the heterogeneity in reads observed in mRNA-seq libraries, due to random fragmentation of the input mRNA, reads obtained from miRNA libraries are very uniform, making quantification methods used in mRNA-seq, such as collapsing reads or fragments per kilobase per million mapped fragments (FPKM), unsuitable for miRNA quantification. To allow quantification of miRNAs, we use adapters containing random sequences (UMIs) to mark each molecule before the amplification step. Using UMIs, we can collapse the sequencing reads similarly to what is done for mRNA-seq [25], obtaining quantitative global sRNA expression data. Another benefit of using UMIs is that it allows an unlimited number of PCR amplification cycles, enabling library preparation from low amounts of starting material, which is especially beneficial for clinical purposes. A UMI reliably reflects molecule counts only if the number of distinct labels is substantially larger than the copy number of the most abundant target molecule; a copy number/labels ratio greater than 0.2 results in an approximate 10–25% undercounting of collapsed reads [26]. In our case, the use of 8-nt-long UMIs resulted in a random barcode pool being saturated for a fraction of highly overexpressed molecules (Additional file 2: Figure S7). Thus, when highly expressed miRNA quantification is needed or a low amount of starting material that requires many rounds of amplification is used, longer UMIs are preferable. Nevertheless, even with 8-nt-long UMIs, reducing the input material by 10-fold produced very similar results (Additional file 2: Figure S4). QsRNA-seq can be adjusted easily to longer UMIs using Table 1. In addition, the use of UMIs was shown to reduce ligation bias as well [10], which we also observed in our samples (Additional file 2: Figure S2). It is also possible to further reduce the ligation bias by mixing several barcodes for a specific sample. Sequencing errors in the UMI sequences were shown to reflect on the accuracy of quantification when using UMIs; therefore, it is also possible to use UMI-tools for the analysis [27]. In this analysis we only aligned our sequences to existing miRNA datasets; however, with the sequencing depth our method provides, it is also possible to use the sequences to identify novel miRNAs by tools such as mirTools [28], ShortStack [29], and miRDeep [30].

The sequencing depth allowed by QsRNA-seq led to a surprising finding of an expressional bias of 23-nt-long miRNAs in the L4 larval stage in *C. elegans*, suggesting a possible connection between the length of miRNA and its role in the organism’s development. Interestingly, a study on endogenous siRNAs in *C. elegans* showed that targets of 23-nt siRNAs are associated uniquely with post-embryonic development [31]. Generation of both endogenous siRNAs and miRNAs is dependent on the Dicer enzyme, and the competition among these processes on resources was suggested to affect development [32]. It is tempting to speculate that sRNA processing by Dicer changes as development progresses, which might be needed for normal development. Studies combining mRNA-seq and sRNA sequencing might be able to shed more light on the function of these 23-nt-long sRNAs.

## Conclusions

After establishing a way to separate very short nucleic acid fragments (shorter than 100 nt) that differ in length by only 20 nt, we developed a new method, QsRNA-seq, to prepare sRNA libraries for HTS. QsRNA-seq is a gel-free, fast, and easy-to-perform method that also utilizes UMI and barcoding, produces high-quality sRNA libraries, and generates high-depth expression data. We discuss why QsRNA-seq will be very useful for sRNA research and for clinical diagnosis. In addition, profiling miRNA in *C. elegans* using QsRNA-seq suggested that not just miRNA expression varies in different developmental stages but also miRNA sizes. We believe that QsRNA-seq can transform the preparation of sRNA libraries into a routine procedure like the preparation of mRNA libraries.

## Methods

### *C. elegans* growth and synchronization

Wild-type *C. elegans* strain Bristol N2 was used in this study and was maintained on OP50-seeded enriched plates at 20 °C as described in [33]. Embryos were isolated from gravid N2 adults by treatment with sodium hypochlorite solution to dissolve animals of all stages but embryos. To obtain synchronized L4 worms, embryos were incubated in M9 media without food at 20 °C for 24 h. Hutched synchronized L1 larvae were grown on OP50-seeded enriched plates at 20 °C until they reached the L4 larval stage.

### RNA extraction

Synchronized embryos or L4 larval worms were washed several times with M9 to avoid contamination with bacteria, and then snap-frozen in liquid nitrogen and ground to powder with a liquid nitrogen pre-chilled mortar and pestle. High molecular weight and low molecular weight RNA fractions were isolated using an miRVana miRNA isolation kit (Ambion). RNA quantity was measured with a Qubit® Fluorometer using a Qubit® RNA HS Assay Kit (Molecular Probes), and RNA quality was estimated using agarose gel electrophoresis and TapeStation (Agilent genomics). Human Brain RNA was obtained from (FirstChoice Human Brain Total RNA, Life Technologies).

### Determining SPRI binding conditions

Volumes of PEG solution and isopropanol were calculated using the equation:

$$ X+\frac{5\mathrm{PV}}{100}+\frac{\mathrm{QV}}{100}=V $$

where *V* is the total volume, *X* is the volume of nucleic acid solution, *P* is the desired concentration (%) of PEG, and *Q* is the desired concentration (%) of isopropanol.

First, a total volume of binding solution (*V*) was calculated by substituting *P*, *Q*, and *X* in the equation for the desired concentrations of PEG and isopropanol and the volume of nucleic acid solution. Next, the volumes of 20% PEG and 100% isopropanol needed for the desired concentrations, equal to 5*PV*/100 and *QV*/100, respectively, were calculated.

To measure the binding efficiency, a solution of 2 ng/μl of synthetic single-stranded DNA oligonucleotide was aliquoted at 50 μl per tube. SPRI beads in 20% PEG (SPRIselect, Beckman-Coulter) and 100% isopropanol were added to each tube at volumes determined using the calculation method above. Size selection was performed according to the manufacturer’s protocol (Beckman’s AMpureXP, left-side selection). Oligonucleotide concentrations in the input and eluted samples were measured with a Qubit® Fluorometer and a Qubit® ssDNA Assay Kit (Molecular Probes). The binding efficiency was calculated by the percentage of the output oligonucleotide obtained from the input quantity.

### Small RNA library preparation

Small RNA libraries were prepared from at least three biological replicas of N2 worms at the embryo or L4 stage. One RNA sample from each stage was selected for preparing two additional libraries, resulting in three technical replicas for each stage.

A step-by-step protocol developed in this study that includes reagents and primers is described in Additional file 1. In short, low molecular weight RNA was ligated to a 5′-adenylated 3′ adapter using T4 RNA Ligase 2, truncated (NEB) in an absence of ATP. For preparation of phosphate-independent libraries after the ligation, the sample was incubated with 5 U of RppH enzyme (NEB) for 30 min at 37 °C. The 3′ adapter-ligated sRNA was separated from free 3′ adapters and longer RNA species and then ligated to a 5′ adapter, containing multiplexing barcode and UMI, using T4 RNA Ligase 1 (NEB). sRNA ligated from both sides was then separated from the adapter dimer and free 5′ adapter to obtain an sRNA library. All the separation steps of the library preparation process were performed using the method described above involving modified SPRI-based size selection of short fragments. The sRNA library was reverse transcribed using a qScript Flex cDNA Synthesis Kit (Quantabio) and amplified using Phusion High-Fidelity DNA Polymerase (NEB). The amplified library was cleaned from primers and irrelevant products below 100 bp and above 200 bp by double-side size selection on SPRI beads (Beckman’s AMpureXP) according to the size-selection conditions recommended by the manual, and its concentration and quality were determined by TapeStation analysis (Agilent Genomics). Libraries prepared by the PAGE-based method were prepared exactly as described in [34]. Libraries were sequenced using 50-bp single-read (SR) sequencing mode on a HiSeq 2500 platform (Illumina). All sequences generated in this study were submitted to the National Center for Biotechnology Information (NCBI) Gene Expression Omnibus (GEO) (http://www.ncbi.nlm.nih.gov/geo/) [35] under accession number GSE96824.

### Sequence processing and expression analysis

The RNA sequences obtained were first de-multiplexed according to the 4-nt barcode. Next, the 3’ adapter sequences were trimmed off by scanning from the 3′ end of the sequence the first instance of the adapter sequence in increments of 1 nt. We then either (1) removed the barcode and UMI (8-nucleotide) and considered these sequences as non-collapsed, or (2) merged identical sequences and then removed the barcode and UMI, and considered these sequences as collapsed.

*C. elegans* sequences were either aligned to the WS220 (Wormbase, www.wormbase.org) genome [36] using Bowtie [37] for size distribution analysis, allowing no mismatches with no more than 10 alignments to the genome (the Bowtie parameters are bowtie -v 0 -e 120 -a --strata --best -m 10) or aligned to miRBase WBcel235 (www.mirbase.org) [38], allowing no mismatches and not more than one alignment (Bowtie parameters are -v 0 -e 120 -a --strata --best -m 1). Human brain sequences were aligned to miRBase GRCh38 [38] with the same Bowtie parameters. We used the full miRBase WBcel235 and not just high-confidence miRNAs to allow a large enough dataset for significant comparison. Size distribution analysis was done on processed sequences before and after alignment to the genome. The DESeq [39] package in R (http://www.r-project.org) [40] was used to evaluate miRNA expression, and the estimateDispersions function in DESeq was used to estimate the dispersion between biological replicas and technical replicas.

## Additional files

Additional file 1: Extended protocol. QsRNA-seq: preparation of sRNA libraries using size selection on SPRI beads. (PDF 196 kb)

Additional file 2: **Table S1.** Sizes of nucleic acid fragments used and generated during QsRNA-seq library preparation process. **Figure S1.** Comparing the yield of PAGE-based method versus QsRNA-seq small RNA library preparation methods. **Table S2**. Comparison between libraries prepared by PAGE-based method and libraries prepared by QsRNA-seq from the same RNA sample**. Figure S2.** Estimation of ligation and PCR derived biases when adding UMI. **Figure S3.** Quantity and purity of QsRNA-seq generated libraries. **Table S3.** Summary of libraries generated in the study. **Figure S4.** Reducing starting RNA quantity by 10-fold produces similar results. **Figure S5.** There is a high correlation between samples generated from the same RNA by PAGE-based method and by QsRNA-seq. **Figure S6.** Variance between collapsed and non-collapsed biological and technical replica samples at *C. elegans* L4 stage is low. **Figure S7.** Collapsing sequences using UMI reduces PCR biases. **Figure S8.** Collapsing sequences using UMI does not change differential expression results of miRNAs. **Figure S9.** The 26-nt-long sequences primarily start with 5’ G. **Figure S10.** Size distribution of *C. elegans* sRNA library sequences with and without UMI. **Figure S11.** Size distribution of *C. elegans* sRNA library sequences generated by the PAGE-based method or by QsRNA-seq. **Figure S12.** Size distribution of sequences from human brain sRNA library generated by QsRNA-seq. **Figure S13.** Size distribution of *C. elegans* sRNA library sequences generated by QsRNA-seq after phosphatase treatment. **Figure S14.** The 22-nt-long microRNAs are expressed similarly in *C. elegans* embryo and L4 stages. (PDF 3458 kb)

Additional file 3: **Table S4.** miRNA expression in *C. elegans* embryo and L4 (biological and technical replicas). (XLSX 125 kb)

Additional file 4: **Table S5.** microRNA expression in human brain sample. (XLSX 89 kb)
