# Supplementary material for: QsRNA-seq: a method for high-throughput profiling and quantifying small RNAs
Source: Genome Biol. 2018 Aug 14;19:113. doi: 10.1186/s13059-018-1495-0 (PMC6090667; doi:10.1186/s13059-018-1495-0)
Supplement: Supplementary file 1 — Extended protocol. QsRNA-seq: preparation of sRNA libraries using size selection on SPRI beads. (PDF 196 kb) [file 13059_2018_1495_MOESM1_ESM.pdf]

QsRNA-seq: Preparation of sRNA libraries using size-selection on SPRI beads.

Protocol outline:

Input: LMW RNA fraction
Ligation of 3'-adapter
Removal of longer RNA species (tRNA, rRNA, mRNA etc.) by size-selection on SPRI beads
Removal of a free 3'-adapter by size-selection on SPRI beads
Ligation of 5'-adapter
Removal of a 3'-5' adapter dimer and free 5'-adapter by size-selection on SPRI beads
Reverse Transcription
Amplification
Library purification by size selection on SPRI beads
Output: small RNA library for Illumina sequencing

RNA extraction

Isolate LMW RNA fraction using column based RNA extraction kits[1]
Measure sample concentration.
Estimate RNA quality by electrophoresis or, better, by Bioanalyser/TapeStation.

Ligation of 3' linker:
We start with 0.5ug of LMW RNA, but much lower quantities can be used.
Combine in an RNase-free microtube:

	Volume (ul)
RNA	X (maximum 12.0ul)
3'-adapter (100uM)	0.5[2]

Mix well by gently pipetting up and down 6-8 times
Incubate 72^0C for 3min
Transfer directly into ice and incubate for at least 1min
Spin down briefly

Add to the sample:

	Volume (ul)
RNAse inhibitor (40u/ul)	0.5
10X T4 ligase buffer	2
50% PEG 8000	4.0
T4 RNA ligase 2 truncated	1.0
H2O (RNAase free)	Up to 20 total
Total	20

Mix well by gently pipetting up and down 6-8 times.
Incubate at 25^0 C for 1h

Removal of 5'-diphosphate (for phosphate independent libs only)
To the ligation reaction add 1ul of RppH enzyme (NEB, 5u/ul)
Incubate at 37^0C for 30 min.

1st SPRI-based size selection

During this step all RNA molecules >60nt are bound to beads and discarded, while adapter-bound -sRNA (39-44nt), free sRNA (19-27nt) and free 3'adapter (18nt) stay in the supernatant

To the 20ul ligation reaction add 40ul H2O to a total of 60ul[3]

Selection Separation Conditions

Add beads and Isopropanol according to the following table

Sample	SPRI BEADS[4] (in 20%PEG)	PEG%	Isoprop.(ul)	Isoprop.(%)	Total Volume
60ul	70[3]	7.5	80	38	210

Mix well by gently pipetting up and down 10 times.
Incubate at room temperature for 5 minutes.
Place the microtube on the magnet stand and allow the beads to settle for 10 minutes or until the supernatant turns completely clear.
Transfer the entire clear supernatant to a new tube

2st SPRI-based size selection

During this step adapter-ligated -sRNA (39-44nt), are bound to the beads, while free sRNA (19-27nt) and 3'adapter (18nt) stay in supernatant and are discarded

To the supernatant from the previous step add beads and Isopropanol according to the table below.

Sample	SPRI BEADS (in 20%PEG)	PEG%	Isoprop.(ul)	Isoprop.(%)	Total Volume
210	90[5]	7.5	150[5]	51	450

Mix well by gently pipetting up and down 10 times.
Incubate at room temperature for 5 minutes.
Place the reaction vessels on the magnet stand and allow the beads to settle for 10 minutes or until the supernatant turns completely clear.
Proceed to **wash & elution**. Elute by adding 19ul H2O

Wash & elution:

1. Remove and discard the clear supernatant.

2. With the microtube still on the magnet, add 600µL of 85% non-denatured ethanol (**freshly prepared** from 100% ethanol) without disturbing the beads and incubate at room temperature for **30** seconds.
3. Remove and discard the ethanol supernatant.
4. Repeat the ethanol wash.
5. Let the beads air dry for 5 minutes. Aspirate residual ethanol and let it dry for additional 2 min. Make sure no residual ethanol is left in the tube before proceeding to elution.
6. Remove the microtube from the magnet stand and add the needed volume of RNAase free water directly on the beads.
7. Gently mix by pipetting 10 times.
8. Incubate at room temperature for 2 minutes.
9. Place the microtube on the magnetic stand and allow the beads to settle for 2 minutes or until the supernatant turns completely clear.
10. Transfer the desired volume of the clear supernatant containing small RNA into a new tube.

Ligation of 5' adapter

Combine:

3' ligated small RNA	18.0
Blocking oligo (100uM)	0.5
5'- adapter	0.5
Total	19.0

Mix by gently pipetting the entire volume up and down 6 –8 times
Incubate at 72^{0}C for 3min, transfer directly into ice and incubate for at least 1min
Spin down briefly

Prepare ligation mix

RNAse inhibitor (40u/ul)	1
T4 RNA Ligase Buffer (NEB)	3
ATP (10mM)	3
T4 RNA ligase1	1
DMSO 100%	3
Total	11

Vortex and spin down
Add 10ul of ligation mix to the tube with the small RNA sample
Incubate at 37^{0}C for 1h
Incubate at **95^{0}C for 30sec** then transfer directly into ice and incubate for at least 60sec
Spin down briefly

3rd SPRI-based size-selection

During this step 3'-5'linked- small RNA (64-72 b) are bound to the beads, while adapter-dimer (45b), and free adapters (18 and 27nt) stay in the supernatant and are discarded

To 30 ul 5'-linked-siRNA sample add 20 ul of RNAse-free H2O to a final volume of 50ul.
Add beads and Isopropanol according to the table below.

5’adapter	Sample	SPRI Beads in 20% PEG (ul)	PEG (%)	Isoprop. (ul)	Isoprop. (%)	Total volume
With UMI (8N)	50	69	7.5	64	35%	170
Without UMI (0N)	50	80	7.5	80	38%	210

Mix well by gently pipetting up and down 10 times.
Incubate at room temperature for 5 minutes.
Place the reaction vessels on the magnet stand and allow the beads to settle for 10 minutes or until the supernatant turns completely clear.
Proceed to **wash & elution** protocol above. Elute by adding 15ul H2O.
Transfer 12.5 ul of the 3'-5’linked- small RNA directly into PCR tube.

Reverse transcription using Quanta qScript Flex[8]

Combine:

3'- 5'-linked siRNA	12.5ul
RT Primer (100uM)	0.5uL
GSPEnchancer	2ul
Total	15ul

Gently pipette the entire volume up and down 6–8 times
Spin down briefly
Transfer to a PCR machine
Incubate at 65^0C for 5 min
Incubate at 42^oC

Add to the sample at 42^0C:

qScript Flex reaction mix (5X)	4ul
qScript reverse transcriptase	1ul
Total	20ul

Gently pipette the entire volume up and down 6–8 times
Incubate at 42^0C for 60min
Incubate at 85^0C for 5 min
Incubate at 10^0C
Proceed to PCR amplification. (Samples can be kept at -20^0C for several days).

Pilot cDNA amplification[9]

Combine in a PCR tube:

	Volume (ul)
RT product	4
H2O	33.0
5X buffer	10
Forward primer (100ul)	0.5
Reverse primer (100uM)	0.5
dNTPs (10mM)	1
Phusion DNA polymerase	1
**TOTAL**	**50**

Perform PCR for 23cycles.
Pause the program after cycles: 14, 17, 20 and withdraw 7ul from the reaction.
PCR conditions
98^0C -30 sec
Cycles 1-4:
98^0C- 10 sec
50^0C- 30sec
72^0C- 15sec
Cycles 5-23:
98^0C- 10 sec
64^0C- 30sec
72^0C- 15sec
72^0C-2min
10^0C-forever

Pilot PCR electrophoresis

Cast 3.0 % agarose/TBE gel
Load 5ul of all the withdrawn samples and the final PCR product
Run at 80V for 1.5h at RT.
Take a picture.
Expected sizes:
8N: library 124-131 bp, Primer-dimer 105bp, artifact 93bp [10]
0N: library 116-124 bp, Primer-dimer 97bp
Evaluate the results and determine the optimal cycle number, which results in clearly visible but not too thick library product band and much weaker or absent undesired product bands.

Main PCR
Prepare PCR reaction as above and amplify for the determined optimal number of cycles.

Cleaning the PCR product on SPRI-beads[11]

Removal of bands >200bp
To 50ul PCR product add 60ul beads (x1.2V)
Mix the total reaction volume by pipetting 10 times
Incubate at room temperature for 5 minutes.
Place the reaction vessels on the magnet stand and allow the beads to settle for 10 minutes or until the supernatant turns completely clear.
Transfer the entire clear supernatant to a new tube

Removal of bands <100bp
To the supernatant add 30ul beads (x1.8V final)
Mix the total reaction volume by pipetting 10 times
Incubate at room temperature for 5 minutes.
Place the reaction vessels on the magnet stand and allow the beads to settle for 10 minutes or until the supernatant turns completely clear.
Proceed according to **wash& elution** protocol above.
Elute in 20ul H2O

Final product validation

Measure product concentration

Validate library purity by Tapstation/ Bioanalyser

The library is ready for HTS sequencing.

Store library at -20^{0}C until use.

Notes

[1]*Column based kits enable non-biased recovery of small RNA. Do not use protocols based on precipitating RNA by isopropanol, as they result in very poor recovery of small RNA. In our hands LMW RNA output of MiRVana kit (Ambyon) resulted in excellent libraries.*

[2] *The adapter must be in a small excess. 1ul of* 100uM (*100pmol*) of 18nt-long 3'-adapter is sufficient for ligating ~700ng (which is a bit less than 100pmol) of pure 22nt oligo. *As most RNA is comprised of longer species, 0.5ul of 100uM 3'-adapter is sufficient for maximal starting quantities. If using lower RNA quantities, dilute the adapter accordingly, as excessive adapter will contribute to adapter-dimer formation.*

[3]*The ligation reaction already contains 4ul of 50% PEG 8000 which equals to 10ul of PEG 20%; to obtain the desired separation conditions the amount of PEG solution added is reduced by 10ul while additional 10ul H2O are added to the sample (obtained sample equals to 50ul RNA in water + 10ul 20% PEG8000).*

[4]*Beckman manufactures two types of SPRI beads, Ampure XP and SPRI-select. SPRI-select is RNAse free certified and has more stringent QA, to ensure reproducibility, but it is much more expensive. In our hands both bead types produced identical results.*

[5] *The sample already contains 80ul PEG and 80ul Isopropanol added during the previous step.*

[6] *By annealing to free 3'-adapter and turning it into dsDNA, the Blocking oligo prevents its ligation to 5'-adapter.*

[7] *To release blocking oligo that might have annealed to 3'-adapter-small RNA to allow its removal by SPRI separation. If not removed, the oligo may compete with a reverse primer for binding site during the RT and PCR reactions (as the oligo is blocked it will generate no product but might compromise the yield).*

[8]*Invitrogen Superscript III or Superscript II can be used for RT instead, however they both are much more expensive and Superscript II results in lower yield.*

[9]*After the system is calibrated, pilot PCR step can be omitted. In this case, PCR is performed for a known optimal cycle number and 5ul of the PCR product is electrophoresed for quality estimation before library purification.*

[10]*An artifact product originates from annealing of reverse primer to one of a random 8N sequences during RT or PCR. It is shorter than primer-dimer as it lacks all/part of 8N and barcode.*

[11] *If primer-dimer band is prominent, we suggest cleaning PCR product by gel-extraction, using* 4% agarose/TBE or, better, *3.0 % BIO-RAD low-range ultra agarose* (*Bio-Rad, Cat. # 161-3107*) */ TBE. Excise the band and weight the excised slice. Extract DNA using gel extraction kit (we use Nucleospin Gel and PCR clean-up kit from Macherey-Nagel). To facilitate dissociation chop the slice finely (use separate razor for each library) and add twice the recommended buffer volume. Elute in 15ul.*

Appendix I- Technical Remarks:

- Aliquot SPRI beads to prevent contamination.
- Conditions for size-selection depend on length of the fragments. If adapters of different size are used, choose suitable conditions using the reference table 1 in the paper.
- In size-selection precision is important, avoid carryover.
- Always use freshly prepared 85% ethanol for size selection and keep the tube closed to prevent evaporation as small RNA may detach from beads at lower ethanol concentration.
- It is very important to immediately transfer the samples to ice after denaturation steps to prevent reformation of secondary structure.
- Aliquot ATP solution. Never thaw ATP in your palm, as it gets degraded.
- Aliquot dNTPs as it gets degraded upon freeze-thaw cycles.
- RT product can constitute no more than 1/10 of PCR reaction volume.
- Use Purple or Orange loading dyes (NEB) as they produce no UV visible bands that may mask you library.
- Use TBE buffer instead of TAE for electrophoresis, as it results in a better resolution of small fragments.

Appendix II- Oligos and reagents

Oligos

3'-adapter	5'-Adenylated and 3'-blocked to prevent oligomerization. rATP at 5' is used by RNA Ligase 2 truncated for ligating the adapter to small RNA. Lack of free ATP in the reaction prevents oligomerization od small RNA
	/5rApp/CTG TAG GCA CCA TCA AT/3ddC/
Blocking oligo	Blocking oligo is reverse compliment to the 3'-adapter. The oligo is blocked from both sides, to prevent formation of undesired ligation products.
	/5AmMC6/ ATT GAT GGT GCC TAC AG/3ddC/
5'-adapters	DNA adapter with 4nt long RNA Barcode on its 3'. 8 random nucleotides (8N) precede the barcode. Has a blocked '5 end to prevent oligomerization.
	/5AmMC6/ ACG CTC TTC CGA TCT NNN NNN NN rArGrCrG<br>/5AmMC6/ ACG CTC TTC CGA TCT NNN NNN NN rCrGrUrC<br>/5AmMC6/ ACG CTC TTC CGA TCT NNN NNN NN rCrUrGrG

	/5AmMC6/ ACG CTC TTC CGA TCT NNN NNN NN rArCrUrU<br>/5AmMC6/ ACG CTC TTC CGA TCTNNN NNN NN rGrGrGrU<br>/5AmMC6/ ACG CTC TTC CGA TCTNNN NNN NN rGrUrUrA<br>/5AmMC6/ ACG CTC TTC CGA TCT NNN NNN NN rUrArUrG<br>/5AmMC6/ ACG CTC TTC CGA TCT NNN NNN NN rUrCrGrC<br>/5AmMC6/ ACG CTC TTC CGA TCT NNN NNN NN rGrCrArG<br>/5AmMC6/ ACG CTC TTC CGA TCT NNN NNN NN rArUrArC<br>/5AmMC6/ ACG CTC TTC CGA TCT NNN NNN NN rUrUrCrU<br>/5AmMC6/ ACG CTC TTC CGA TCT NNN NNN NN rCrArArU<br>/5AmMC6/ ACG CTC TTC CGA TCTNNN NNN NN rArArGrA<br>/5AmMC6/ ACG CTC TTC CGA TCTNNN NNN NN rUrGrArA
RT oligo	PCR reverse primer is also used as RT primer to prevent generation of an undesired PCR products primed by RT primer, which are compatible with Illumina sequencing.
PCR Forward primer	5'-adapter flanked by Illumina sequences<br>GAT ACG GCG ACC ACC GAG ATC TAC ACT CTT TCC CTA CAC GAC GCT CTT CCG ATC T
PCR Reverse primer	Reverse complementary to 3'-adapter + Illumina sequences<br>CAAGCAGAAGACGGCATACGAATTGATGGTGCCTACAG

Reagents:

miRVana miRNA Isolation Kit
Applied Biosystems (Ambion) Cat. # - AM1560 (w/ phenol)

RiboLock RNase Inhibitor
ThermoFisher Scientific Cat. # EO0381

T4 RNA ligase 1(ssRNA Ligase)
NEB Cat. # M0204S

T4 RNA ligase-2, truncated
NEB Cat. # M0242L

QScript Flex cDNA synthesis kit
Quanta, cat # 95049-025

Phusion High-Fidelity DNA Polymerase
NEB Cat. # M0530S

Agencourt Ampure XP
Beckman Coulter Cat. # A63881
OR
SPRI select reagent Kit
Beckman Coulter Cat. # B23319

DynaMag-2 magnetic stand
ThermoFisher Scientific Cat. # 12321D
