# Supplementary material for: QsRNA-seq: a method for high-throughput profiling and quantifying small RNAs
Source: Genome Biol. 2018 Aug 14;19:113. doi: 10.1186/s13059-018-1495-0 (PMC6090667; doi:10.1186/s13059-018-1495-0)
Supplement: Supplementary file 2 — Table S1. Sizes of nucleic acid fragments used and generated during QsRNA-seq library preparation process. Figure S1. Comparing the yield of PAGE-based method versus QsRNA-seq small RNA library preparation methods. Table S2. Comparison between libraries prepared by PAGE-based method and libraries prepared by QsRNA-seq from the same RNA sample. Figure S2. Estimation of ligation and PCR derived biases when adding UMI. Figure S3. Quantity and purity of QsRNA-seq generated libraries. Table S3. Summary of libraries generated in the study. Figure S4. Reducing starting RNA quantity by 10-fold produces similar results. Figure S5. There is a high correlation between samples generated from the same RNA by PAGE-based method and by QsRNA-seq. Figure S6. Variance between collapsed and non-collapsed biological and technical replica samples at C. elegans L4 stage is low. Figure S7. Collapsing sequences using UMI reduces PCR biases. Figure S8. Collapsing sequences using UMI does not change differential expression results of miRNAs. Figure S9. The 26-nt-long sequences primarily start with 5’ G. Figure S10. Size distribution of C. elegans sRNA library sequences with and without UMI. Figure S11. Size distribution of C. elegans sRNA library sequences generated by the PAGE-based method or by QsRNA-seq. Figure S12. Size distribution of sequences from human brain sRNA library generated by QsRNA-seq. Figure S13. Size distribution of C. elegans sRNA library sequences generated by QsRNA-seq after phosphatase treatment. Figure S14. The 22-nt-long microRNAs are expressed similarly in C. elegans embryo and L4 stages. (PDF 3458 kb) [file 13059_2018_1495_MOESM2_ESM.pdf]

Figures and Tables

	No UMI barcode (0N)	8nt-long UMI barcode (8N)
small RNA	19-27(22)	19-27 (22)
3'-adapter	18	18
5'-adapter	19	27
3'-ligated small RNA	37-45 (40)	37-45 (40)
3',5'-ligated small RNA	56-64 (59)	64-72 (67)
Adapter-dimer	37	45
**Amplification products:**		
Final library	116-124 (119)	124-132 (127)
Adapter-dimer	97	105

Table S1. Sizes of nucleic acid fragments used and generated during QsRNA-seq library preparation process.

* Numbers in parenthesis correspond to predominantly miRNA-based libraries; for example, human brain library.

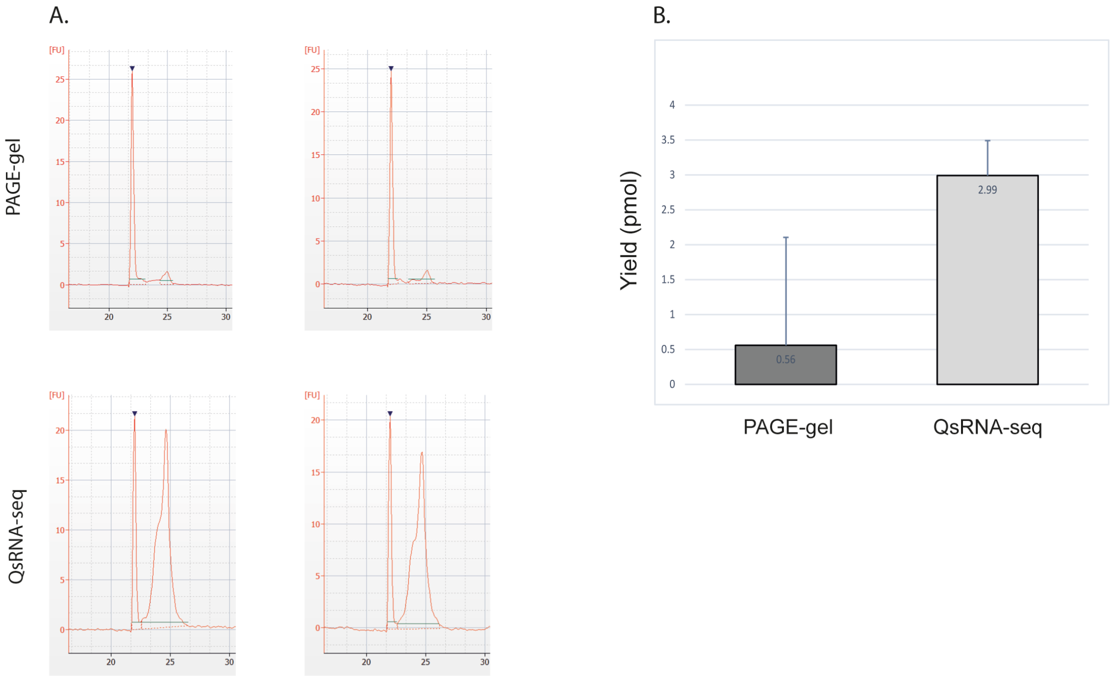

Figure S1. Comparing the yield of PAGE-gel versus QsRNA-seq small RNA library preparation methods. Two technical replicas of small RNA libraries were prepared from 20pmol synthetic 22nt-long RNA oligo using either QsRNA-seq or PAGE-gel based method. 3'-5' ligated RNA product was diluted 1/10 and analyzed on Agilent 2100 Bioanalyser using Eucaryote total RNA pico kit. A. Electropherogram traces. Upper row- PAGE-gel based

libraries, lower row- QsRNA-seq libraries. The left peak marked by black arrowhead represents a 25nt marker, the right peak is the generated library. B. Bar plot of average yield of libraries, including standard deviation. The yield of the QsRNA-seq method is not only higher but also highly consistent.

Sample barcode	Preparation method	Total sequence reads	Aligned reads	%rRNA	%tRNA	%snoRNA
0N_CTGG	PAGE-gel	7623764	6116334 (80.23%)	1597256 (20.95%)	22296 (0.29%)	10024 (0.13%)
0N_ACTT	PAGE-gel	926369	810633 (87.51%)	235528 (25.42%)	4373 (0.47%)	2866 (0.31%)
0N_AAGA	PAGE-gel	580914	482891 (83.13%)	137721 (23.71%)	3322 (0.57%)	1074 (0.18%)
0N_CTGG	QsRNA-seq	51970925	42851297 (82.45%)	22706563 (43.69%)	505348 (0.97%)	596686 (1.15%)
0N_ACTT	QsRNA-seq	29885440	24257612 (81.17%)	16311371 (54.58%)	135976 (0.45%)	163469 (0.55%)
0N_AAGA	QsRNA-seq	46147169	39252298 (85.06%)	21315099 (46.19%)	795336 (1.72%)	605211 (1.31%)
8N_AAGA	QsRNA-seq	50598663	39705176 (78.47%)	10518346 (20.79%)	323607 (0.64%)	310581 (0.61%)
8N_CTGG	QsRNA-seq secondary	38890616	32926625 (84.66%)	8546620 (21.98%)	372758 (0.96%)	129107 (0.33%)

Table S2. Comparison between libraries prepared by PAGE-gel and libraries prepared by QsRNA-seq from the same RNA sample.

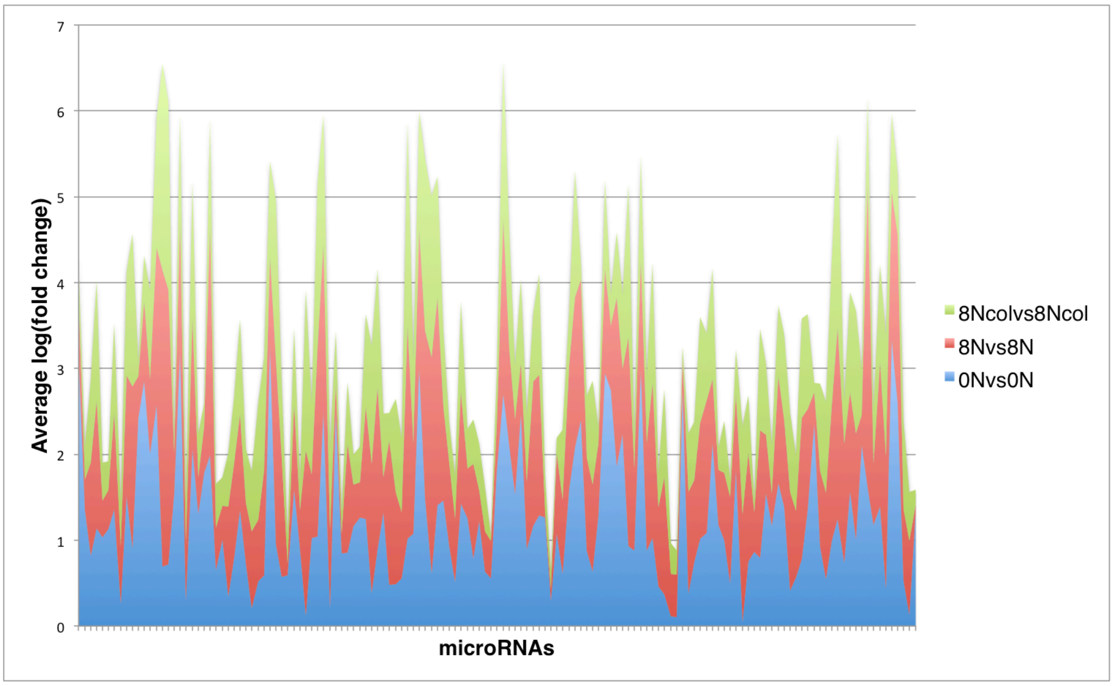

Figure S2. Estimation of ligation and PCR derived biases when adding UMI. The average differential expression of miRNAs between samples with different barcodes was evaluated without UMI (0Nvs0N), with UMI (8Nvs8N) and with UMI after collapsing (8Ncolvs8Ncol). The results of the different comparisons are presented by stacked graphs. X-axis are single miRNAs and the Y-axis is the average of the log(fold change) comparing the different barcodes. miRNAs with less than 5 counts were discarded from the analysis.

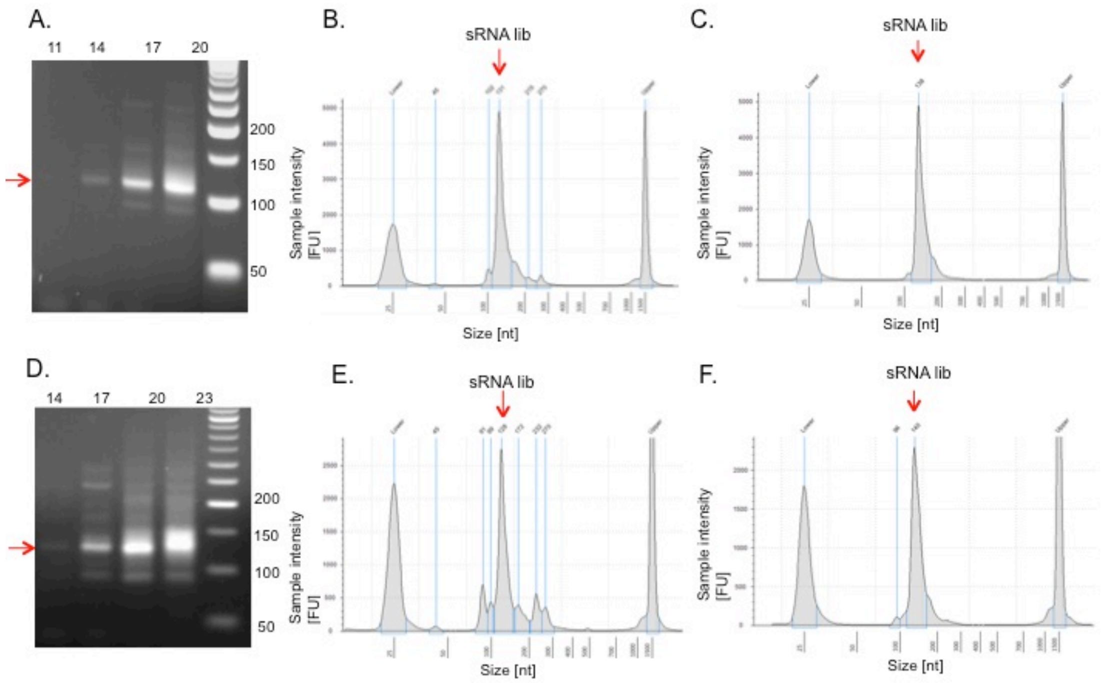

Figure S3. Quantity and purity of QsRNA-seq generated libraries

Libraries prepared from 1µg LMW RNA fraction extracted from *C. elegans* L4 stage (upper panel A-C) and 1µg of Human Brain Total RNA (lower panel D-F). (A,D) 3% agarose gel electrophoresis of 5 µl amplification product after cycles 11,14,17 and 20 (A) and cycles 14,17,20 and 23 (D). sRNA Library product (indicated by red arrow) is clearly visible at cycle 11(A) and cycle 14 (D). (B,E) Tapstation traces of the amplification products after 17-cycles. (C,F) Tapstation traces of the sRNA library after purification of the 17-cycle amplification product by SPRI double size selection. sRNA library corresponding peaks are marked by red arrows. Peak sizes are indicated in blue; the outer peaks titled "Lower" and "Upper" are 25nt and 1500nt molecular size markers, respectively.

Library Name in GEO	UMI-8N	RNA Source	Bio replicate Num	Tech replicate Num	RNA input (ug)	Total sequence reads	Aligned reads	Alignment %	8N-Collapsed reads
N2_E_PR_B1_T1_8N	yes	Embryo	1	1	1	3.10E+07	2.84E+07	91.62%	1.36E+07
N2_E_PR_B1_T2_8N	yes	Embryo	1	2	1	2.36E+07	2.12E+07	89.74%	7.82E+06
N2_E_PR_B1_T3_8N	yes	Embryo	1	3	1	1.79E+07	1.57E+07	87.44%	8.55E+06
N2_E_PR_B2_8N	yes	Embryo	2		1	3.52E+07	3.04E+07	86.24%	1.40E+07

N2_E_PR_B3_8N	yes	Embryo	3		1	1.24E+08	1.10E+08	88.70%	2.03E+07
N2_L4_PR_B1_T1_8N	yes	L4	1	1	1	2.05E+07	1.65E+07	80.46%	5.12E+06
N2_L4_PR_B1_T2_8N	yes	L4	1	2	1	6.14E+07	4.69E+07	76.46%	1.56E+07
N2_L4_PR_B1_T3_8N	yes	L4	1	3	1	4.61E+07	2.78E+07	60.38%	1.40E+07
N2_L4_PR_B1_0.1ug_8N	yes	L4	1		0.1	5.88E+06	3.65E+06	62.05%	2.19E+06
N2_L4_PR_B2_8N	yes	L4	2		1	3.62E+07	2.20E+07	60.68%	1.45E+07
N2_L4_PR_B3_8N	yes	L4	3		1	1.17E+08	9.83E+07	84.11%	2.12E+07
N2_E_PR_B1_T1_0N	no	Embryo	1	1	1	2.39E+07	2.03E+07	85.29%	NA
N2_E_PR_B1_T2_0N	no	Embryo	1	2	1	3.59E+07	3.06E+07	85.27%	NA
N2_E_PR_B1_T3_0N	no	Embryo	1	3	1	3.37E+07	2.80E+07	83.10%	NA
N2_L4_PR_B1_T1_0N	no	L4	1	1	1	2.82E+07	1.89E+07	66.96%	NA
N2_L4_PR_B1_T2_0N	no	L4	1	2	1	3.35E+07	1.80E+07	53.91%	NA
N2_L4_PR_B1_T3_0N	no	L4	1	3	1	3.10E+07	2.18E+07	70.38%	NA
HumBr_8N	yes	Human brain			1	3.65E+07	NP	NP	7.33E+06

Table S3. Summary of libraries generated in the study.

NA- non applicable, NP- not processed.

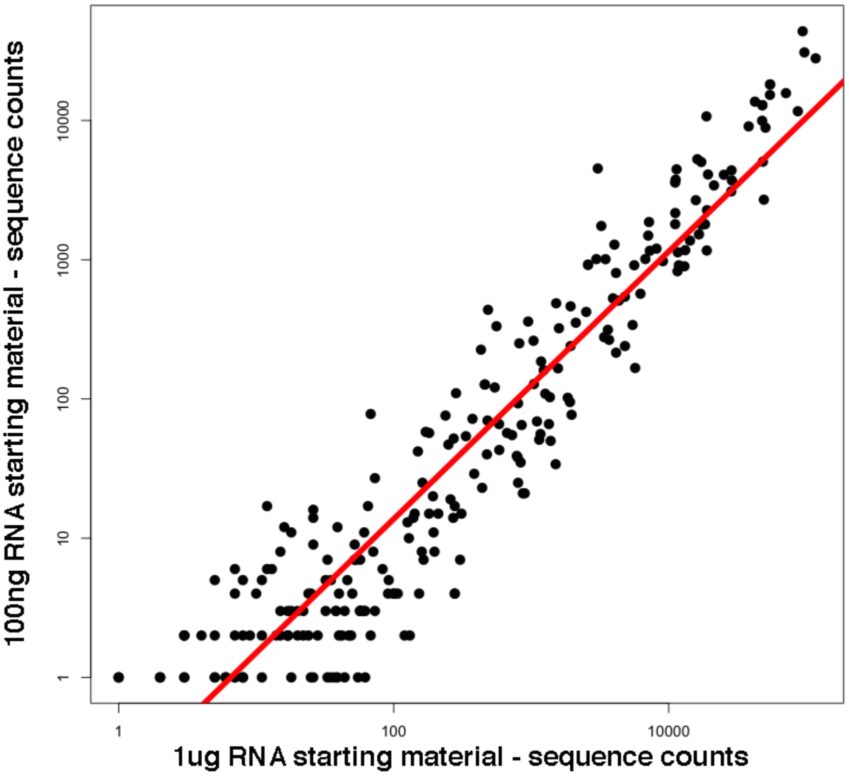

Figure S4. Reducing starting RNA quantity by 10 fold produces similar results. Log scale plot comparing miRNA expression from two libraries constructed using either 1ug or 100ng from the same input material from L4 larval stage. Every dot in the plot represents a sequence count for a miRNA after sequences were collapsed based on 8N UMI. The red line is the regression line for all miRNAs presented in the graph. The graph shows a linear result

suggesting that reducing input material does not affect expression results. R^2=0.9 by Pearson correlation with p-value < 2.2e-16.

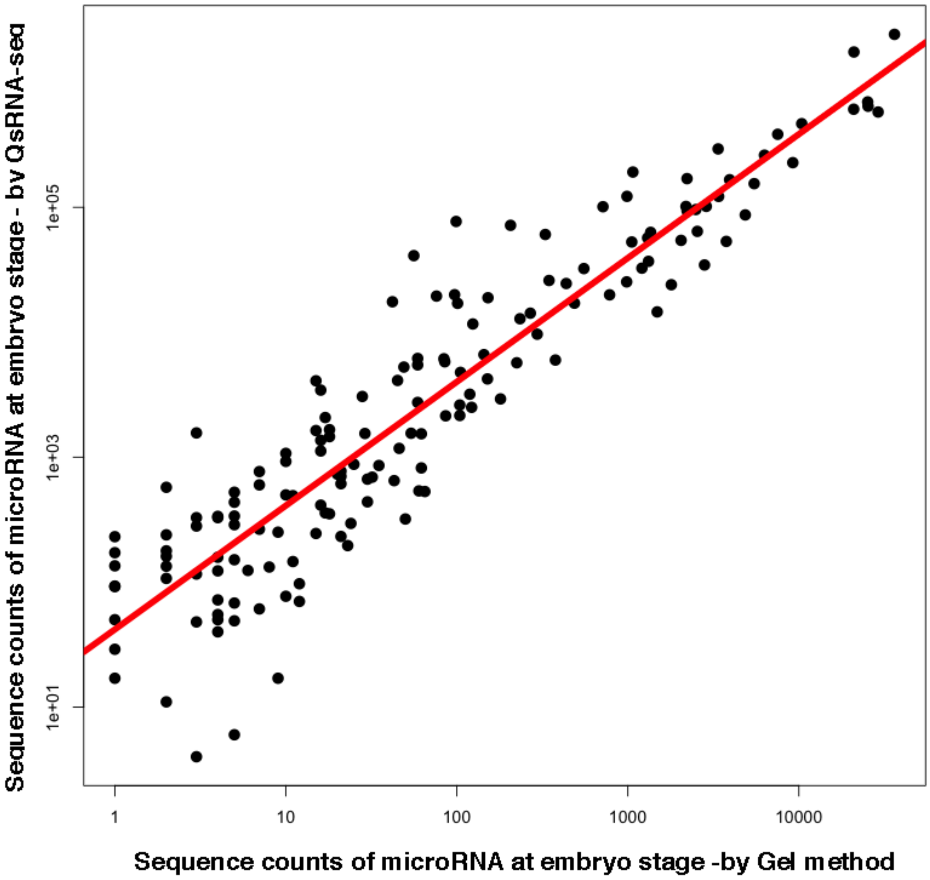

Figure S5. There is a high correlation between samples generated from the same RNA by PAGE-gel method and by QsRNA-seq. Log scale plot comparing miRNA expression from two libraries constructed by PAGE-gel (x-axis) and QsRNA-seq (Y-axis) from the same input material from *C. elegans* embryo stage. Every dot in the plot represents a sequence count for a miRNA. The red line is the regression line for all miRNAs presented in the graph. R^2=0.88 by Pearson correlation with p-value < 2.2e-16.

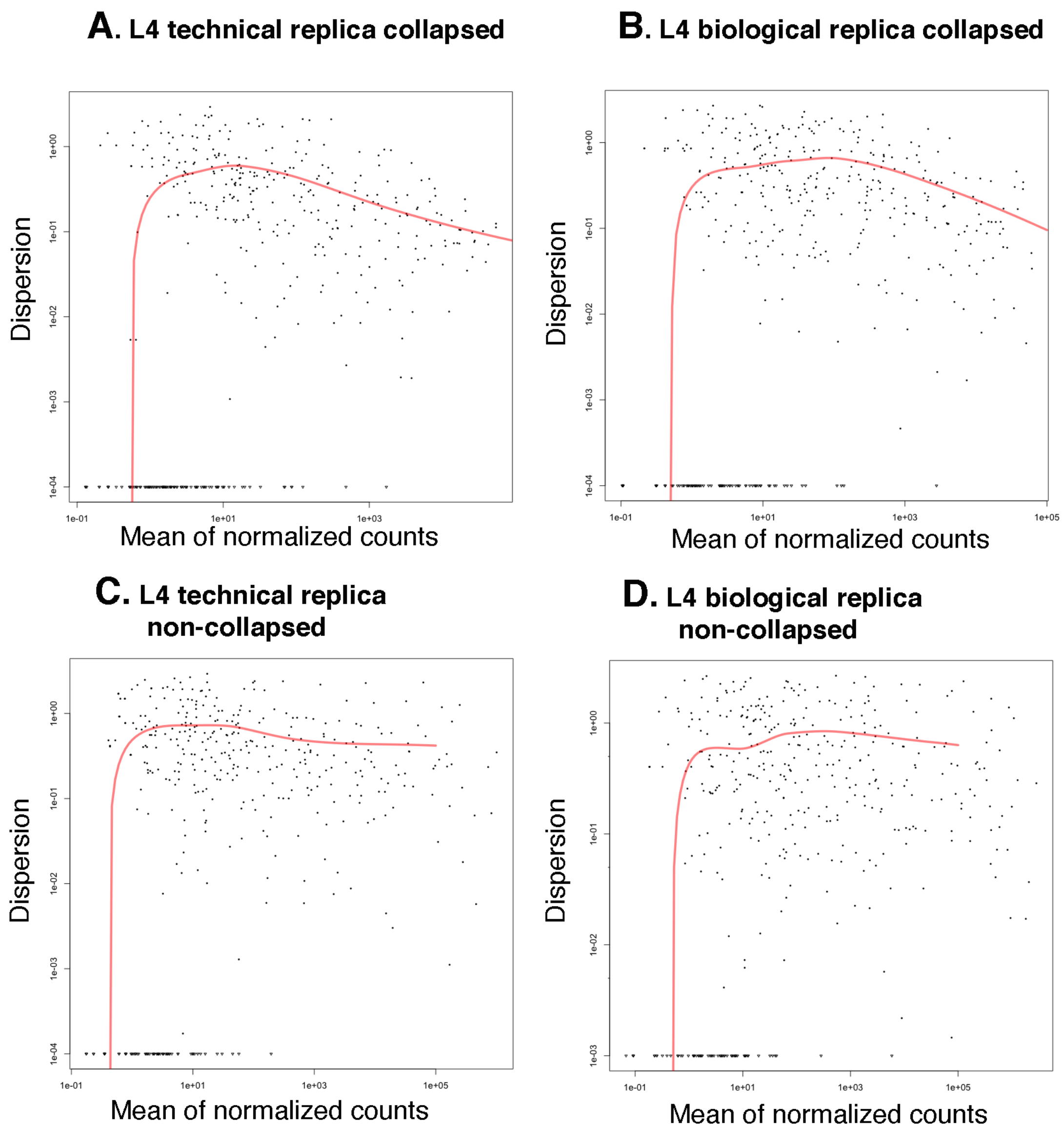

Figure S6. Variance between collapsed and non-collapsed biological and technical replica samples at L4 stage is low. Dispersion plots generated by DESEQ package in R using estimateDispersions function. Sequences aligned to each miRNA were counted and the variance of the three replica samples was estimated. Each dot in the plot represents variance between the replica samples for specific miRNA counts. X-axis represents the mean normalized counts. Y-axis represents a dispersion value, which is the variation between samples squared. All plots are samples generated from L4 developmental stage, (A) technical replica samples dispersion estimated with collapsed reads, (B) biological replica samples dispersion estimated with collapsed reads, (C) technical replica samples dispersion estimated with non-collapsed reads, and (D) biological replica samples dispersion estimated with non-collapsed reads.

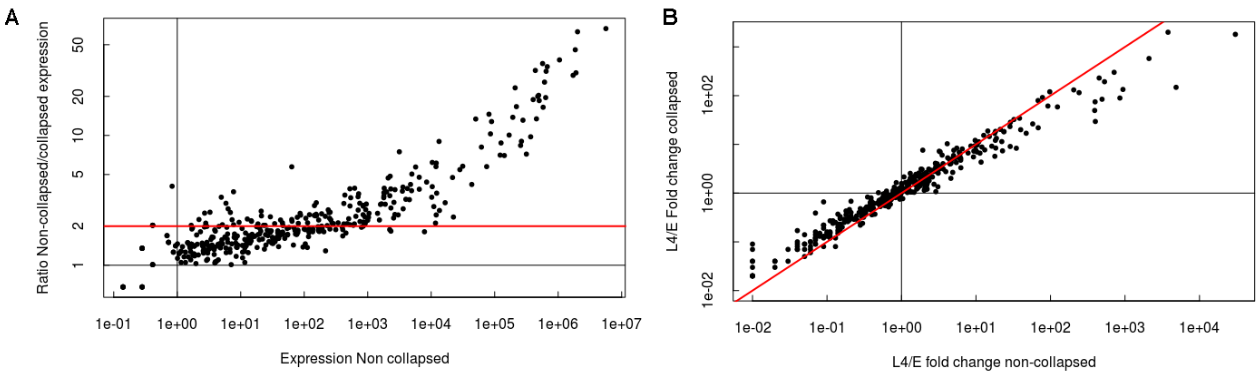

Figure S7. Collapsing sequences using UMI reduces PCR biases. (A) X-axis is the normalized mean count at embryo stage when not using UMI (e.g. non-collapsed), Y-axis is the ratio between the non-collapsed and collapsed normalized mean count at embryo stage. Each dot represents a miRNA. The plot shows that the non-collapsed to collapsed ratio is not constant and is dependent on the level of miRNA expression. While for low expressed miRNA (up to ~100 non-collapsed reads) the ratio is less than two, for more abundant miRNA the un-collapsed reads are over amplified by PCR, resulting in a significantly higher ratio. Red line is Y=2 for emphasis. (B) A plot presenting the differential expression data (fold change) of miRNAs between L4 and embryo developmental stages calculated using non-collapsed reads (X-axis) and collapsed reads (Y-axis). Each dot represents a miRNA. While for miRNAs exhibiting low expressional changes between L4 and embryo (up to 10 fold change in expression), expression fold change calculated using collapsed and non-collapsed data are comparable, for miRNAs exhibiting high expressional changes between the two stages the difference in fold change obtained by collapsing and non-collapsing is significantly larger. Red line is Y=X for emphasis.

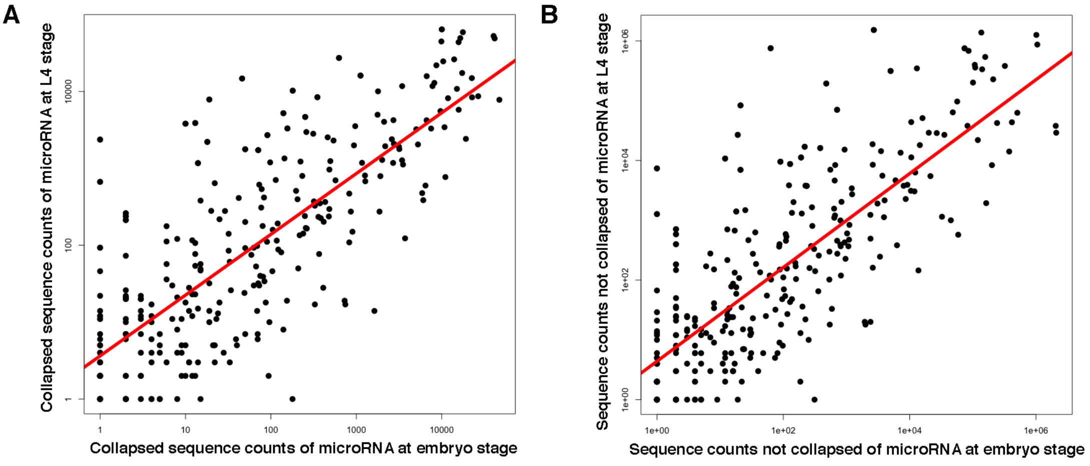

Figure S8. Collapsing sequences using UMI does not change differential expression results of miRNAs. Log scale plot comparing miRNA expression in embryo stage versus L4

larval stage when using UMI (A) or not (B). Every dot in the plot represents a sequence count for a miRNA. The red line is the regression line for all miRNAs presented in the graph. Sequences obtained from the two libraries were either collapsed, e.g. same sequences with identical UMI were considered as one sequence (A) or were left as is (B). It is apparent that while collapsing sequences significantly reduces the number of counts obtained for each miRNA by removing PCR amplification products, it does not affect L4/Embryo differential expression (i.e., whether a miRNA is upregulated or downregulated)

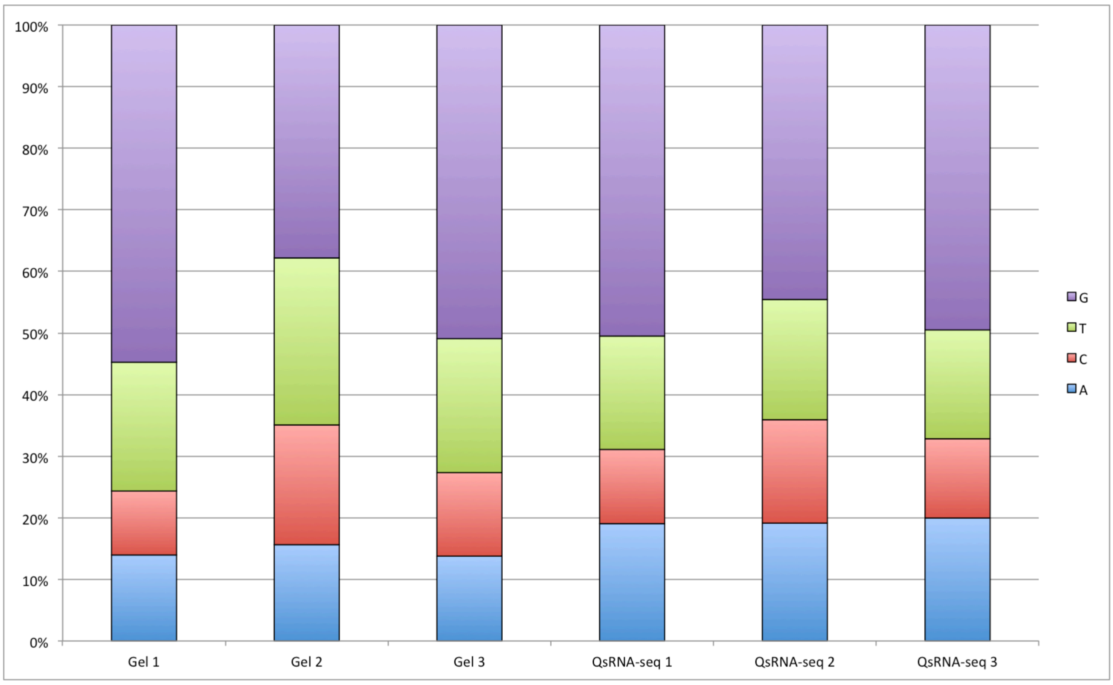

Figure S9. 26nt long sequences primarily start with 5'G. Bar graph presenting the distribution of the first nucleotide of 26nt long sequences generated by PAGE-gel (Gel1-3) or QsRNA-seq (QsRNA-seq 1-3) methods.

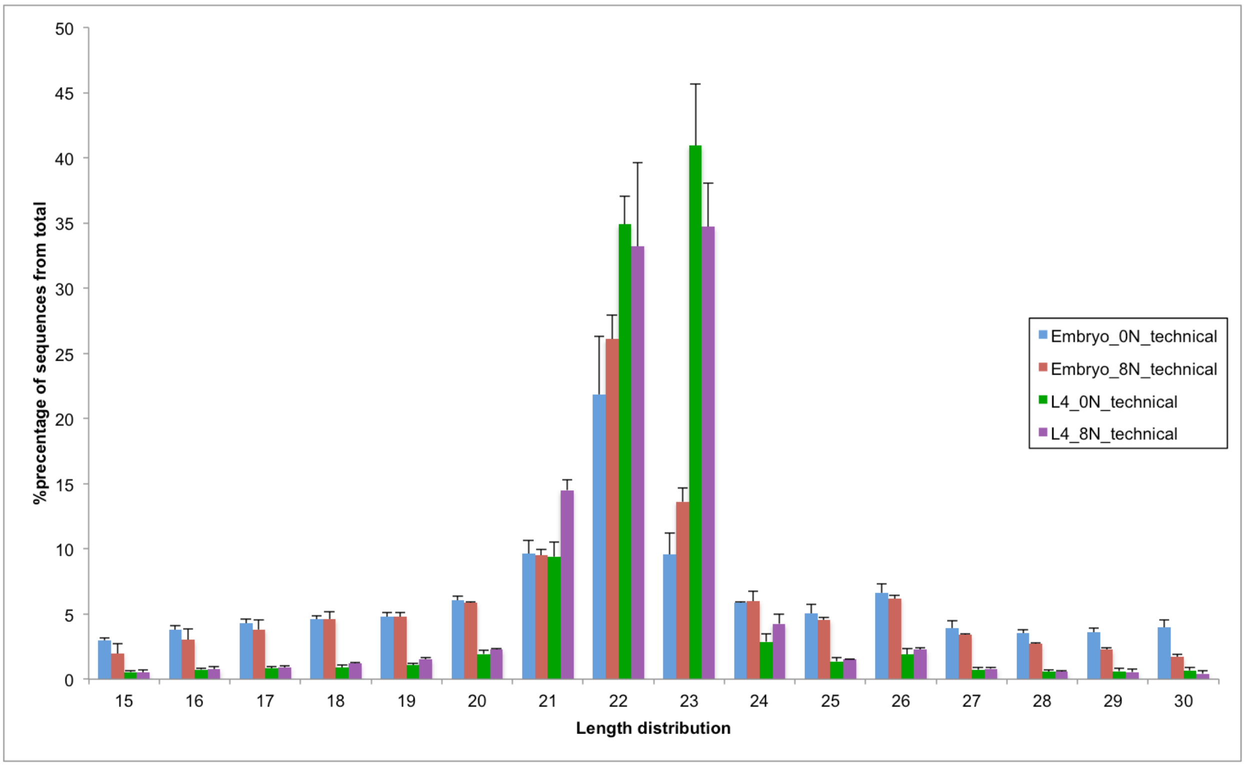

Figure S10. Size distribution of *C. elegans* sRNA library sequences with and without UMI. Bar chart presenting the percentage of sequences from 15nt to 30nt long. Percentage of each sequence length is calculated from the total number of sequences in this size range. Sequences are from embryo (blue and red bars) or from L4 developmental stage (green and purple bars) samples, without UMI (0N, blue and green bars) or with UMI (8N, red and purple bars). Each sample represented is the average of three technical replicas. Standard deviation is also presented.

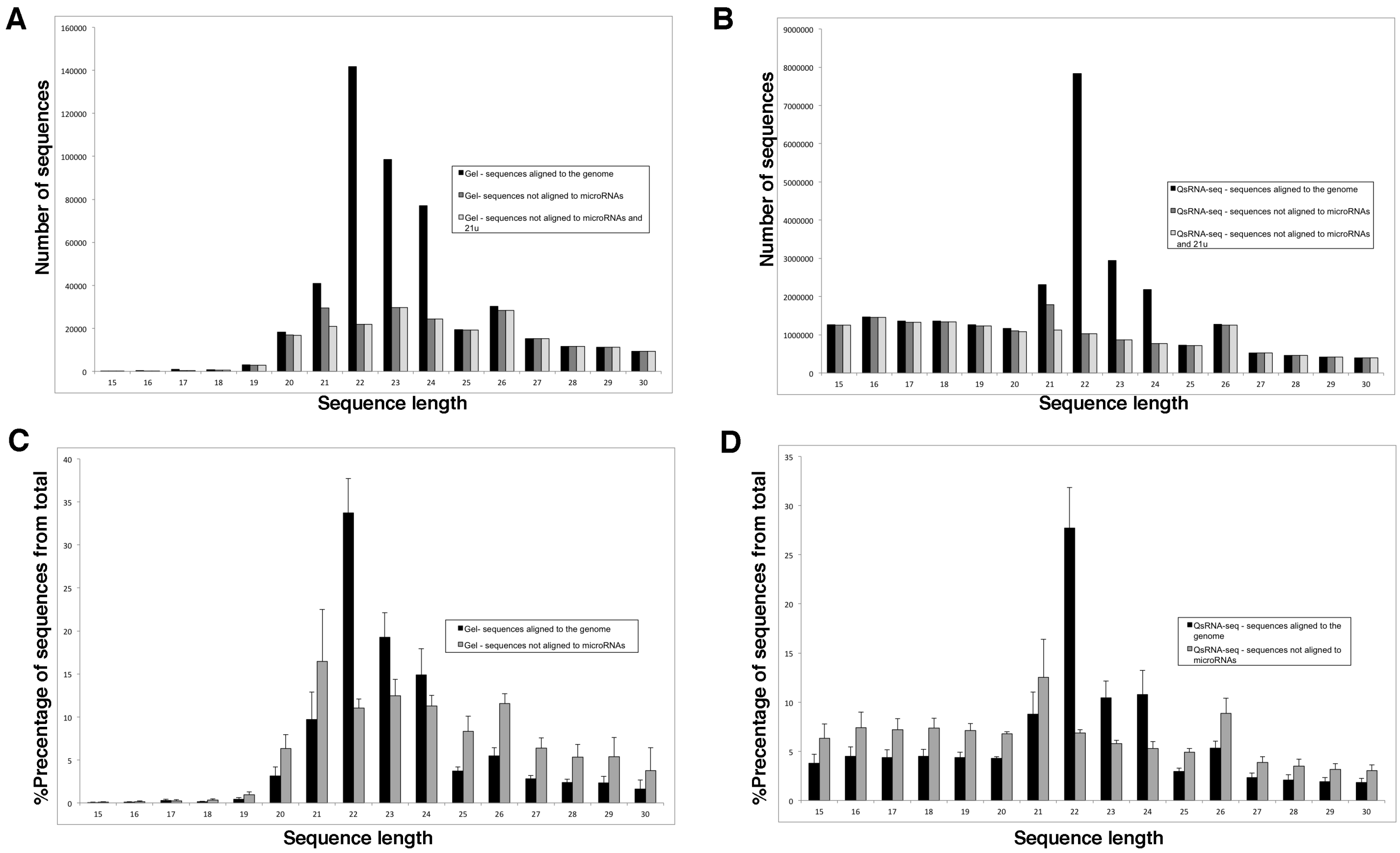

Figure S11. Size distribution of *C. elegans* sRNA library sequences generated by PAGE-gel or by QsRNA-seq (A,B) Bar charts presenting number of sequences for each sRNA sequence length from 15nt to 30nt. The sequences are from one sample from embryo stage generated by PAGE-gel (A) or by QsRNA-seq (B) from the same RNA. (C,D) Bar graphs presenting percentage of sRNA for each sequence length from 15nt to 30nt from total number of sequences. Average of 3 technical replicates generated by PAGE-gel (C) and average of 3 technical replicates generated by QsRNA-seq (D). All samples were generated from the same RNA without UMI. Standard deviation is also presented. Black bars represent sequences that align to the genome, dark grey bars are sequences that did not align to miRNAs from sequences presented in the black bars, light grey bars represent sequences from the dark grey bar that also did not align to 21U sRNAs.

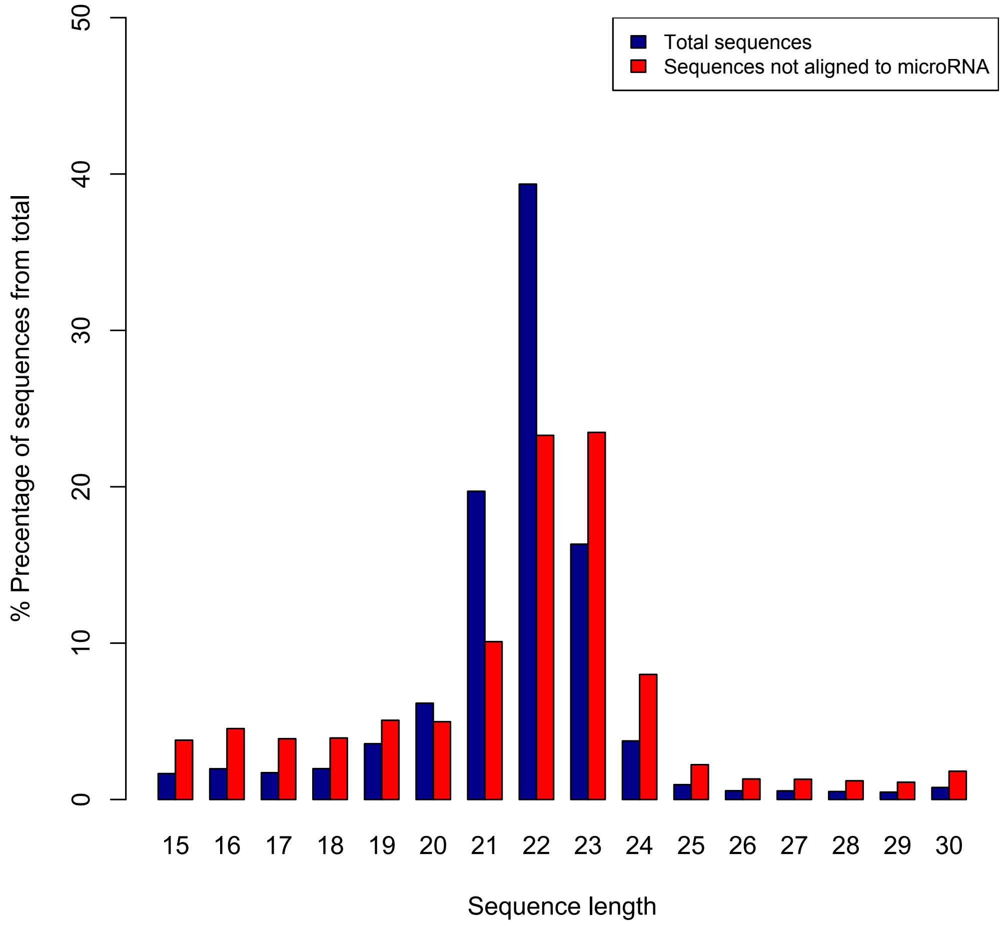

Figure S12. Size distribution of sequences from human brain sRNA library. Bar chart presenting the percentage of sequences from 15nt to 30nt long. Percentage of each sequence size is calculated from the total number of sequences at this size range. Blue bars represent raw sequences, and red bars are sequences that did not align to miRNAs. As expected, most miRNAs in the human brain sample are 21-22nt long.

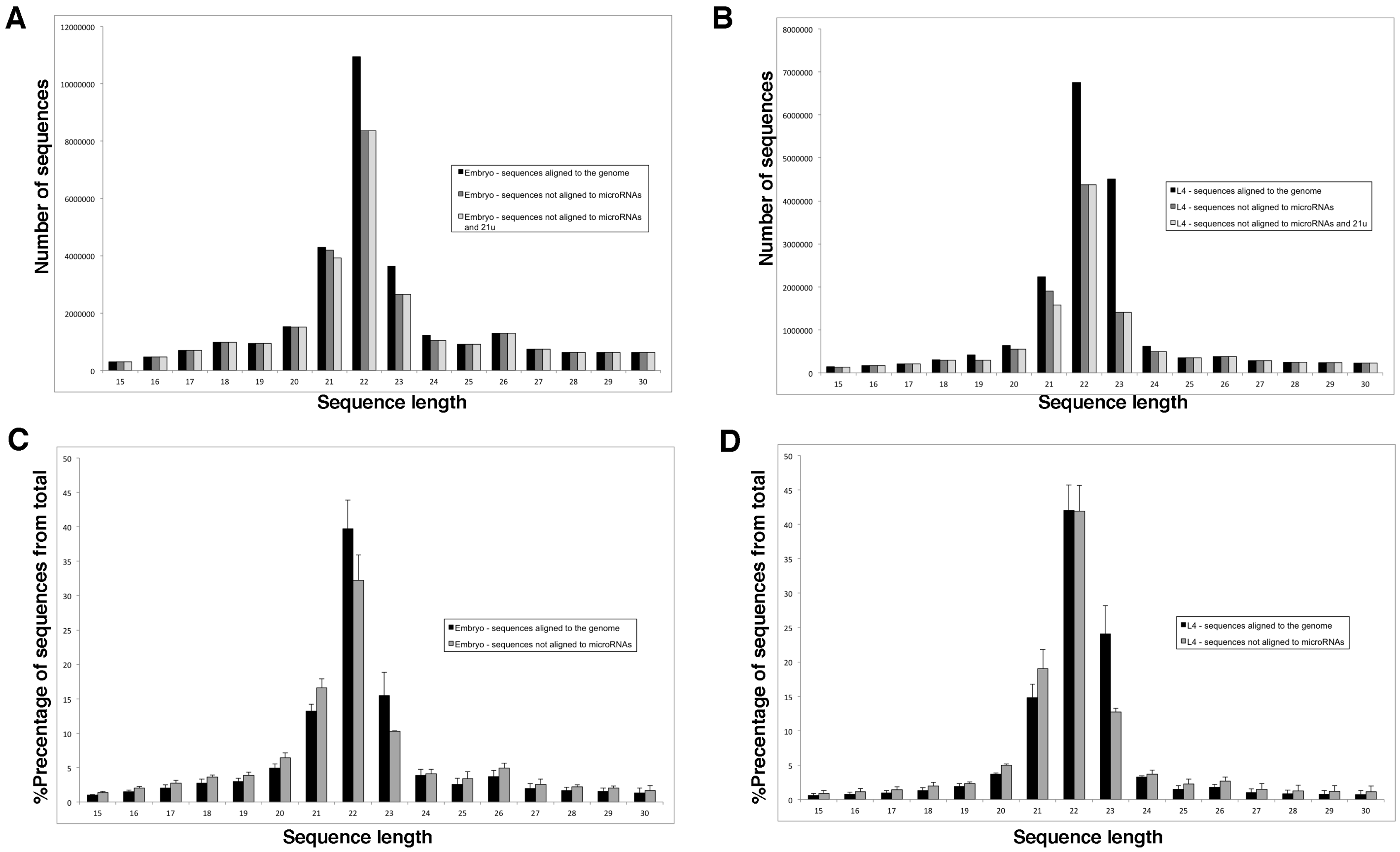

Figure S13. Size distribution of *C. elegans* sRNA library sequences generated by QsRNA-seq after phosphatase treatment ((A,B) Bar charts presenting number of sequences for each sRNA sequence length from 15nt to 30nt. The sequences are from one sample from embryo stage (A) or L4 larval stage (B). (C,D) Bar graphs presenting percentage of sRNA for each sequence length from 15nt to 30nt from total number of sequences. (C) Average of 3 biological replicates at embryo stage. (D) Average of 3 biological replicates at L4 larval stage. Standard deviation is also presented. Black bars represent sequences that align to the genome, dark grey bars are sequences that did not align to miRNAs from sequences presented in the black bars, light grey bars represent sequences from the dark grey bar that also did not align to 21U sRNAs.

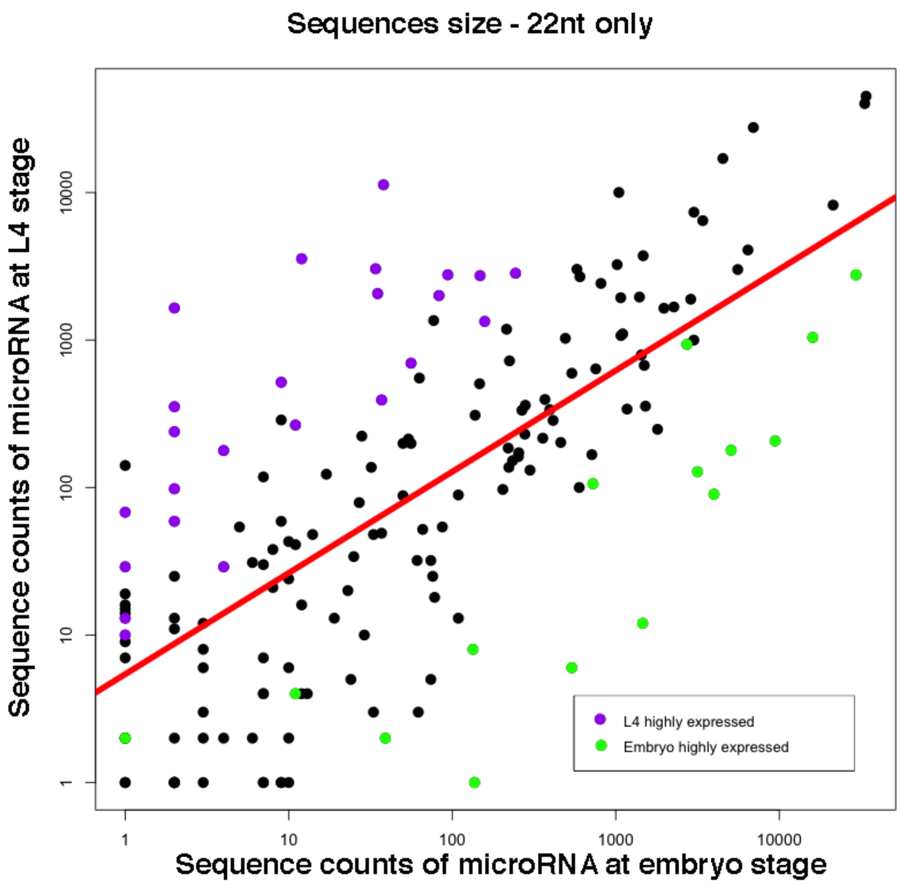

Figure S14. 22nt long microRNAs are expressed similarly in embryo and L4 larval stage.

Log scale plot comparing 22nt long microRNA expression between Embryo and L4 developmental stages. Every dot in the plot represents a miRNA. Green dots indicate miRNAs predominantly expressed at embryo stage; purple dots indicate miRNAs predominantly expressed at L4 stage. The red line is the regression line for all miRNAs presented in the graph.
